# Part II. Analecta

**Published:** 1827-01

**Authors:** 


### PRACTICAL MEDICINE;

BEING

### The Spirit of the Medical Journals,

*Foreign and Domestic;*

WITH COMMENTARIES.

### PART II.

### ANALECTA.

" Ore trahit quodcunque potest, atque addit acervo."

### 1. DISEASE OF THE HEART.

In the last number of the Medico-Chirurgical Review, page 606, allusion was made to the case of Mr. P. (a half-pay quarter-master) residing in King Street, Westminster, as labouring under "organic disease of the heart, with its usual consequences, effusion, &c. &c." as ascertained by auscultation. This patient died suddenly in the night of the 14th October, just two months after the above diagnosis was given, and one month after it was published. We examined the body in the presence of Dr. G. P. Morris, Mr. Ollier, Surgeon of the Western Dispensary, Mr. Lavies, Mr. Clapton, and Mr. Henry Johnson. The following were the appearances:—Œdema of the lower extremities—two or three pints of water in the abdomen—right cavity of the chest filled with clear serum, the lung of that side being shrivelled to about twice the size of the fist. No marks of inflammation in that side of the thorax. In the left side nothing but an immense pericardium could be seen, on first inspection, the lung being quite concealed. The pericardium was thickened by successive layers of coagulable lymph, and agglutinated to the sternum in front and to the ribs on the side. When opened, a heart of most enormous dimensions presented itself. The apex of the heart was firmly glued to the pericardium by a strong and ancient adhesion, the size of a shilling. The parietes of the left ventricle were *one inch and three quarters*, in some places, thick—the cavity of the ventricle in proportion. The mitral valve appeared shrivelled and greatly out of proportion to the other parts in the neighbourhood—it could not be made to cover the auriculo-ventricular opening, and was therefore quite imperfect in its function. The auriculo-ventricular opening was of great calibre, so that the two chambers were almost one cavity with an imperfect valve intervening. The left auricle was dilated, proportionally with the ventricle, but its parietes not proportionally hypertrophied. The parietes of the right ventricle were not half an inch in thickness, but the chambers on this side were enlarged greatly. The semilunar valves at the origin both of the aorta and pulmonary artery, were unaltered; but the origin of the aorta itself was rather widened, so that the semilunar valves, at this part, could not have completely shut the root of the aorta, when the ventricle was in a state of diastole. Scales of bone covered the root of the aorta, which was only enlarged for about the space of two inches from the heart. The vessel, from that point, possessed its natural calibre. The heart was computed to weigh about two and a half, or three pounds.

In the abdomen, marks of extensive chronic peritonitis every where existed. The convolutions of the intestines were glued together in many places, and to the reflected peritoneum. The stomach was glued to the liver and to all the contiguous parts. The liver itself was small and condensed, and its peritoneal covering whitened with coagulable lymph. Its structure was granite-looking, and a large white tubercle, an inch and a half in diameter, was cut through, in making an incision into its parenchymatous structure. The gall-bladder was so agglutinated to the neighbouring parts, especially to the colon and stomach, that we had some difficulty in bringing it into view. It contained a number of small angular calculi, as black as jet. They changed colour, however, on being heated, and were evidently the common biliary calculi. The coats of the gall-bladder were much thickened; but the state of the ducts could not be properly ascertained amid the mass of adhesive inflammation in which they were entangled. The stomach was very large, and its mucous membrane extensively discoloured, nearly black in many places, and a considerable portion softened and quite abraded. We had not time to examine the mucous membrane of the intestines. There was nothing else remarkable.

We think the foregoing case is interesting, as verifying the diagnosis which was given two months previously, by aid of auscultation and percussion, in the presence of several medical gentlemen, especially Dr. Marshall Hall, Dr. Hewett, Dr. Macann, Mr. Lavies, and others. This diagnosis, as respected the organic disease of the heart and the effusion in the chest, the two principal features of the disease, could not have been given, with any thing like confidence, except through the aid of percussion and auscultation. The action of the heart was perfectly regular, and the pulse rather small, to the last; nor did the patient ever complain of much pain or uneasiness in the region of the heart. In fact, his attention was directed to symptoms in almost every part of the body, except the spot where the fatal disease was situated. Mr. Parker had led rather an irregular life, as a soldier, and had been intemperate till lately. He dated the origin of his complaint in November, 1825, when he had been straining rather violently while brewing some ale. He then felt a sudden pain beneath the right scapula, near the shoulder, which continued for several months. Next followed dyspnœa on going up stairs, with sudden feelings of suffocation, causing him to start up in bed. Pains in the epigastrium and region of the liver succeeded, with a sense of beating at the pit of the stomach. After these symptoms had continued for a few months, he got into a state of great irritability and irascibility, complaining of such a host of strange and anomalous feelings in various parts of the body, that he was set down by more than one or two of his medical attendants as completely hypochondriacal. Dropsical effusions, however, in the lower extremities compelled the physicians who took this view of the case, to fear that there was something more than fancy in the business. The biliary secretion was depraved—the appetite irregular—the digestion imperfect. Then, of course, the digestive organs were considered in fault, and mercurials and bitters were employed, as well as diuretics; but these produced no permanent benefit, and the poor man was driven almost to insanity by the horrible feelings of mind and body. Still the disease of the heart was not suspected, though the state of the breath, the paucity of urine, and œdema of the lower extremities, led to the belief that hydrothorax was impending. Such was the state of the case when the writer of this article was consulted, on the 26th of June last, nearly five months before the fatal termination. A single application of the ear detected the organic disease of the heart. The pulsation could be heard even in the right side of the chest; but the most remarkable phenomenon was a whizzing or purring noise in the region of the heart, corresponding exactly with the ventricular contractions, or, in other words, with the pulse. This led, at once, to the conclusion, that there was disease, or at least imperfection, in the valvular apparatus of that side of the heart, while the extent of pulsation left no doubt of the enlargement of the organ. The chest sounded very badly in the region of the heart, and no respiratory murmur could be heard in the lower three-fourths of the *right* side of the chest. These phenomena were verified by numerous auscultations, and Dr. Marshall Hall confirmed them by two separate examinations, in the month of August following. Neither Dr. Hall nor Dr. Hewett, however, were quite satisfied respecting the nature of the disease of the heart, though convinced that there was hydrothorax of the right side in particular.

It would be interesting to trace the origins of the various organic changes which this unfortunate officer presented on dissection, but no regular or minute history of the symptoms could be obtained, in consequence of the afflicted patient's running from one medical man to another in the vain hope of cure. The reporter, however, saw him every day, for nearly five months, and never had reason for altering the opinion formed during the first interview. There can be little doubt, that the enlargement of the heart was the primary disease. The adhesions and thickening of the pericardium bore all the marks of long standing inflammation; and the band which united the apex of the heart with the internal surface of the pericardium, was as white, dense, and strong as ligament. We conceive, then, that the enlargement of the heart had been going on *for years;* and that all the other changes were of a far posterior date. The peritoneal inflammation agglutinating the abdominal viscera, though not recent, was, in all probability, much more so than that in the chest. The effusion of water into the right side of the thorax was, unquestionably of recent formation, and had gradually increased during the attendance of the reporter. We all know how soon a lung will be diminished to a very small compass, when encroached upon by water in the chest. One morbid structure, however, which dissection disclosed in this case, may perhaps claim seniority over the enlargement of the heart. That was, the granulated texture and diminution of bulk in the liver. We think there can be little doubt that this morbid change was produced by the early and long continued habits of irregularity, not to say intemperance, of a soldier's life. It is such a change as we not unfrequently see resulting from similar habits. But the question is, had this change of structure in the liver any thing to do with the organic disease of the heart? We think it is not at all improbable that the liver disease, attended, as it must have been, with disordered function in the digestive organs generally, contributed originally to disturb the function of the heart, and ultimately its structure. But this was anterior to the illness which caused the patient's death, and had nothing to do with that event. Neither could any human sagacity have detected organic change in the liver during life. Its *function* was deranged, it is true, but so it often is, without any change of structure. The organic disease of the heart, in fine, produced the dropsical effusion in the chest—and this was the immediate cause of death. The adhesive inflammation in the abdomen was an accidental occurrence, and although its sequelæ occasioned much distress, they did not affect life.

2. CAN THE BLOOD BE THE SEAT OF DISEASES?*

That the fluids, as well as the solids, do occasionally undergo alterations in a state of disease, is pretty generally admitted; but the question is, can the blood be the primary seat of diseases—or can a change in the condition of this fluid be entitled to the denomination of diseases? and can disordered function in various organs be the consequences of this change in the fluids? M. Segalas thinks that an answer in the affirmative may be safely given. Numerous experiments have now been made on animals, with the view of ascertaining this point, by different physiologists. The following suite of trials were confined to the effects of alcohol and nux vomica on dogs.

1. If a certain quantity of alcohol be thrown into the jugular vein of a dog—half an ounce, for example—the animal is instantly killed, and the blood is found very much changed, being grumous, and, in colour, resembling milk that is turned. The lungs are reddened, and firmer than natural.

2. If the alcohol be diluted by four or five parts of water, the animal soon falls down, in a state of complete intoxication, being motionless, insensible, and only exhibiting signs of life by slow breathing carried on by the abdominal muscles, and by a scarcely perceptible pulse.

3. When the quantity of alcohol is small, as for example a drachm, the animal only staggers about for a few minutes, and soon after appears as if nothing had happened. This return to health takes place in proportion to the elimination of the alcohol, as is evident from its odour on the pulmonary exhalation.

4. The above experiment was several times repeated, and always with the same result. If the animal be watched, and the experiment repeated as soon as the pulmonary vapour ceases to exhale the odour of the alcohol, an ounce of this spirit may thus be passed through the veins of a dog, of 30 pounds weight, in the course of an hour.

5. The same quantity thrown at once into the veins, quickly suppresses the respiration, and the heart ceases to pulsate in the course of two or three minutes.

On examination of the body, no alteration can be perceived in the solids, with the exception of some increase of density in the lungs. But the blood was always found to be materially altered. It was not grumous, as after the injection of the pure alcohol; but it had a creamy appearance, and was more uniformly thickened.

*Exp.* 6. If the alcoholic injection be made into the bronchia, the inebriation takes place equally soon when introduced into the veins, and the appearance of the blood is the same in both cases, but, in the former, a larger quantity of alcohol is necessary to induce these changes in the blood, and a longer time for its operation, than in the latter mode of experimenting.

7. Whether we divide the eighth pair of nerves, or leave them intact, injection of alcohol into the bronchia produces the symptoms of intoxication in the same time, and to the same degree.

8. Intoxication through the medium of the stomach requires more alcohol, and much longer time for its operation, than when the fluid is thrown into the venous or respiratory vessels. The intoxication is also much more slow in being dissipated.

9. The blood appears thickened after this kind of inebriation, even when the animal is killed by asphyxia.

* Experiences relatives à cette question, "La sang peut-il être siège de Maladies? Memoire lu à l'Académie des Sciences, 21 Feb. 1826. Par Segalas D'Echapare. Archives, Septembre, 1826.

10. The effects of alcoholic injection in the stomach were the same, whether the pneumo-gastric nerves were divided or not, provided the œsophagus was tied to prevent vomiting.

11. Alcoholic injection into the cavity of the pleura, peritoneum, the bladder, and the subcutaneous cellular tissue, acted in the same manner; but with a quickness and intensity proportioned to the absorbing powers of the parts.

12. In these different cases, whether the stomach be extirpated or not, the effects of alcoholic injections are the same.

13. In proportion as the mass of blood was diminished by previous venesection, it required less alcohol to produce intoxication, and more time for the dissipation of its effects.

14. The injection of a good deal of water into the veins produces a different effect—intoxication then requires a greater quantity of alcohol, and a shorter time for dissipation.

15. The quantities of alcohol being equal, it requires a shorter time to produce intoxication, by the medium of the stomach, after a great loss of blood—and a longer time after much aqueous injection into the veins.

These facts, says our author, are in conformity with what we see in men habituated to alcoholic drinks, or to breathe an alcoholic atmosphere; and prove that the condition necessary for intoxication, is the presence of a certain quantity of alcohol in the blood.

They prove (*Exp.* 1.) THAT concentrated alcohol produces a chemical action on the blood in a living state. THAT diluted alcohol (*Exp.* 2, 6, 8, 11,) determines immediate inebriation, if injected into the veins or bronchia—and the same, with more or less rapidity, if injected into other parts. THAT the effects of aclcohol injected elsewhere than into the veins, are in proportion to the intensity and celerity of the absorbing function of the part, and altogether independent of the nerves distributed on those parts, particularly of the nerves of the stomach. See *Exp.* 6, 7, 8, 10, 11, 12. THAT these effects are retarded or accelerated by such circumstances as restrain or favour absorption. THAT intoxication is dissipated at the time the alcohol is expelled from the circulation; and more or less quickly, according to the circumstances which are more or less favourable to exhalation. THAT the effects of alcohol are intense, not in proportion to the quantity brought in contact with the organs, but with the quantity absorbed into the blood. Finally, THAT extreme intoxication and death from intoxication coincide with a manifest alteration in the blood itself, and a slight alteration in the solids.

Is it then to be doubted that alcoholic intoxication is owing to the presence of this fluid in the blood, and that the phenomena which accompany it are occasioned by the abnormal action of the blood thus changed on the organs, and especially on the nervous system? What, in fact, are the phenomena of intoxication, but the expression of an altered state of the blood—*the symptoms of a disease of the blood?*

These circumstances will explain some facts which were before inexplicable—such as the influence of oil in preventing the effects of alcohol—and that of ammonia and acetate of ammonia in dissipating those effects. Oil retards absorption, while ammonia and its acetate favour the elimination of alcohol from the circulation. Perhaps, too, these last-mentioned substances have some effect on the nervous system, or some power of neutralising the effects of alcohol on the blood.

Our author next proceeds to a suite of experiments with the alcoholic extract of nux vomica, in which, if we substitute the word tetanus for intoxication, we shall have the same results as in the first series of experiments. In several of these experiments care was taken to innervate the whole, or certain parts of the body, by section of the spinal marrow, &c. in order to shew that the action of the nux vomica on the muscles was determined by its presence in the blood, and not by its influence on the nervous system. Thus, for example, when the spinal marrow was divided in the dorsal or cervical regions, the poison injected or absorbed into the veins produced tetanic rigidity in the parts paralyzed less quickly, but more permanently than in the muscles which were not deprived of their nervous communication with the brain or spinal marrow.

The experiments here related, observes our author, authorize us to conclude, that tetanus produced by the nux vomica has, for the primary condition of its development, the presence of the poison in the blood, and that the phenomena which accompany it, are owing to the abnormal action of this fluid on the nervous system.* Can it be then denied that these phenomena are the indices of an altered state of the blood?—" *the symptoms of a disease of the blood?*"

These experiments, our author observes, explain, in some measure, the action of nux vomica on paralyzed limbs in man, and why its action is more permanent on the paralyzed muscles than on those which are in health. The communication of these last with the brain not being cut off, enables them sooner to shake off the influence of the poison.

There was one remarkable phenomenon appeared in these experiments which ought not to be passed over in silence, namely that when a moderate quantity of nux vomica was injected into the blood, the tetanic spasms take on a regular intermittent character, ceasing and re-appearing at stated periods. This is a very curious phenomenon, and, perhaps, it may one day help to explain the intermittent types of those fevers dependent on the introduction of a miasmal poison into the system.

---

### 3. REGENERATION OF SPHACELATED URETHRA.†

This case deserves to be ranked among the marvellous, and Dr. Brown, we hope, will excuse us if we appear somewhat sceptical on the present occasion.

*Case.* A man, 50 years of age, had evacuated his urine with considerable difficulty for two years, and during that period, was in the habit of passing " a *small* metallic sound into the urethra." On the 14th May, he was found labouring under violent inflammation of the scrotum, penis, and the whole of the hypogastrium, with swelling and tension along the line of the urethra. Nevertheless, the urine still continued to pass through the regular channel. The inflammation continued unmitigated till the 17th, when fluctuation was perceptible on the left side of the abdomen. An incision was made, and " a tea-cupful of abominably fetid pus discharged." On the 19th, the tumefaction had much subsided, though the discharge was profuse, and extensive sloughs had formed. On the 20th, the greater part of his urine flowed through the wound—and in the evening, it all came through this channel. On the 23d, a large slough was drawn out from the wound, four inches in length, " comprising full three inches of the urethra." This extensive slough is preserved in spirits, and a drawing of it is given.

---

* The experimenter means, of course, the action of the poisoned blood on the *nerves of the muscles themselves*, as well as on the general nervous system.—*Rev.*

† Dr. Brown. Ed. Jour. Med. Science, No. 4.

An examination of the drawing does not lead us to believe that it comprises any portion of the urethra; and a perusal of the subsequent history of the case confirms our scepticism. On the 5th June, or 12 days after this event, the man emptied his bladder by a full stream through the urethra. On the 23d of the same month the man was walking about, averring that—"he made water as well as ever he did in his life, none having passed through the wound in the abdomen *since the 5th instant*."

The above case needs little comment. The man ruptured the membrane of the urethra by means of his "small metallic sound." Some urine was extravasated, and caused the suppuration, and formation of horribly fetid pus. A slough was drawn out from this wound, with a canal in it, or something like a canal, which has been mistaken for the urethra. Had it really comprised "three inches" of that canal, the patient would not have been walking about in three or four weeks, "making water as well as ever he did in his life." The test of miracles is the experience of man; and we apprehend that the experience of man is against "the regeneration of a urethra" in the time and manner above-mentioned.

---

### 4. SOME RARE CASES IN SURGERY, BY PROF. SCARPA.*

Although not very partial to extraordinary cases either in physic or surgery, yet all such cases are worthy of record, since they may prove landmarks or beacons, on future occasions, where we are in doubts and difficulties.

### Enormous Collection of Milk in the Mamma.

*Case.* 1. A young female peasant, of small stature, but robust constitution, perceived, ten days after a second accouchement, a considerable swelling in the left arm-pit not preceded by any inflammatory symptom, or pain. She continued to suckle her infant from both breasts—but more especially from the left, in the hope of dissipating the swelling; but the effect was quite contrary to her expectations, for the flow of milk from that breast daily diminished, while the tumour in the axilla increased. This tumefaction gradually extended to the mamma, and at last occupied the whole of it. In the course of two months the breast got to such a size that it measured 34 inches in circumference, and, when sitting, the mamma rested on the thigh of that side. The skin covering this tumour presented no particular alteration in appearance, except that it was rather tense and shining, the subcutaneous veins being dilated. Professor Scarpa thrust a middle-sized trocar into the tumour near the axilla, where the integuments appeared thin. A flow of pure milk followed, and ten pints were drawn off in a continued stream. The celebrated Dr. Frank was present at this operation, and was not a little astonished. When the milk was evacuated, the mamma was scarcely more voluminous than the other. The wound made by the trocar was enlarged, and a tent introduced, for the purpose of facilitating any subsequent discharge. The milk was sent to M. Scopoli, professor of chemistry, and its analysis shewed that, notwithstanding the long sojourn of the fluid in the breast, it differed in none of its physical or chemical properties from the recently secreted milk of a female.

The obliteration of the cavity where the milk was lodged could not be effected till a seton was established there. The cure was ultimately complete.

---

* Opusculi di Chirurgia, vol. ii.

### A Foreign Body in the Rectum.

*Case* 2. A young peasant, habitually constipated, experienced, at one time, so much inconvenience, that he introduced into the rectum a reed-stalk, for the purpose of mechanically assisting the evacuation of the hardened fæces. It had the desired effect; but in the second introduction, the reed-stalk escaped from his grasp, and went up beyond his reach. All the straining and violent efforts which he made to eject the foreign body were in vain, and he was soon in great agony. He was carried the next day to the hospital, where the surgeon on duty made some fruitless attempts to extract the offending body. He then gave him a purgative. The succeeding day Scarpa saw the patient, and, on careful examination of the abdomen, discovered the piece of reed in the *right* iliac region, where the least pressure occasioned great pain. In the evening of the same day the extremity of the reed was found to have stretched over towards the *left* iliac region, shewing that the foreign body had a tendency to pass up the colon.* In the course of the night it got into the sigmoid flexure, and by the next day, when Scarpa made his rounds, the reed had got into the transverse arch of the colon, and was lodged just under the ensiform cartilage of the sternum. Scarpa thought it would now soon reach the cæcum; but by five o'clock in the afternoon it had retrograded to the hollow of the left ilium, where it was very prominent. Scarpa now placed the patient in the position for lithotomy, and introduced a very flexible gum-elastic catheter, the size of the ring-finger, till its point reached the sigmoid flexure of the colon. The wire of the catheter was then introduced to keep it steady. By means of the fingers of one hand on the abdomen, and by manœuvring the point of the catheter with the other, he managed to bring the reed into a position parallel with the line of the gut, whereas it appeared to him to be lodging obliquely across the passage. By thus keeping it in a proper position, and pressing gently on its upper extremity, through the parietes of the abdomen, he enticed the reed down within reach of the finger in the rectum, and then easily extracted it with a pain of forceps.

The Professor remarks that this case leaves no doubt of the *anti-peristaltic* motion of the colon—a fact which indeed has long ago been proved by the disappearance of injections from that gut, and by their being vomited by the mouth.

The above case bears considerable similitude to the case related some 17 years ago by Mr. Thomas, of Leicester Place, and with which most of our readers, perhaps, are acquainted. The patient was a gentleman habitually costive, and who was in the habit of daily introducing a piece of flexible cane, the size of a finger, into the rectum, and there leaving it till the nisus for evacuation took place. One day, however, it escaped out of reach. Nevertheless the motions passed regularly, and it was seven days after the accident, when Mr. Thomas saw him. He was then labouring under symptoms of abdominal inflammation, and was in great distress. No part of the cane could be felt per anum, but one end of it was very evident, projecting midway between the ileum and umbilicus on the *right side*. The part was extremely sensible. An anodyne enema was first administered, and two hours afterwards Mr. Thomas was enabled gradually to introduce

* There seems to be some obscurity here. One would have expected that the first appearance of the foreign body would have been in the *left* iliac region, in its way to and through the sigmoid flexure of the colon. It is probable, however, in this case, that the rectum lay more towards the right than the left side of the sacrum.

the hand into the rectum, and in 20 minutes extracted the cane. It was nine inches in length, and one of its extremities very ragged and uneven.

The venerable Professor relates, in the same volume, two cases of sanguineous tumours removed, one from the upper lip, the other from the palatine arch. The former was a nævus maternus, the latter had been growing for a great many years. Considerable hæmorrhage succeeded the ablation of these tumours, but was arrested by pressure and styptic applications.

---

5. MODIFICATION OF SORE THROAT IN CHILDREN.*

Dr. Hamilton, Jun. had thrown out some hints respecting this modification of sore throat in the last edition of his work, on the Management of Children, in the following words :—

" 'There is a very dangerous, but fortunately rare, modification of sore throat, which begins in the form of a whitish spot, like that of thrush (though more definite in its shape, being round or oval,) on one or both tonsils, unaccompanied at first by fever, and attended with only a trifling degree of uneasiness in swallowing. By and by this spot enlarges, its edges become of a florid colour ; fever steals on ; and the act of swallowing grows painful. A slough gradually forms, with evident ulceration of its edges ; the fever increases ; and headache and restlessness supervene.

" 'The partial separation of the slough, together with the rosy colour of the edges of the ulcer, with the moderate degree of fever for some days, promise a favourable issue. But very unexpectedly slowness of breathing, without either difficulty or wheezing, takes place, with excessive and sudden sinking of the living powers; and it generally happens, that, within a day from this change, the fatal event takes place. The breathing at first falls to eighteen respirations in the minute; then to sixteen; to twelve; and finally to ten or eight. Sometimes, with the sloughing, the tonsil swells; and, in some cases, both tonsils are affected.

" 'Hitherto, with one exception, this disease has proved mortal in every case in which the author has been consulted; and he considers the slow breathing to be a sure symptom of the fatal termination.

" 'With respect to the nature of the disease, his experience is too limited to enable him to give a decided opinion. He has repeatedly known two individuals of the same family attacked in succession with the disease ; but he does not feel warranted in pronouncing it to be infectious.

" 'It is with feelings of sincere regret, that he has to state, that no mode of treatment, yet discovered, seems to have any influence in checking the progress of this disease. The most powerful local applications, such as stimulating gargles, the use of caustic, &c. and all the ordinary means of supporting the strength, have, in the cases to which the author was called, been pursued with much anxiety and activity, without any benefit. The operation of opening the windpipe, was, in one interesting case, where, from the swelling of both tonsils, there was apparently a mechanical obstruction to the breathing, had recourse to without any avail.' "

Further experience has taught Dr. H. that two other symptoms occasionally attend this fatal disease—offensive fetor of breath—sudden occurrence of cynanche trachealis. The former symptom has generally appeared from six to nine days after the disease had commenced; but the latter symptom has taken place earlier, viz. before the sixth day.

---

* Dr. James Hamilton, Jun. Ed. Journ. Med. Science, No. 4.

Our author concludes, therefore, that this curious affection proves fatal in two ways—by exciting cynanche trachealis—and by inducing slow respiration and sinking of the vital powers. "In repeated instances, these two different terminations have occurred in the same family." The modus operandi of the former termination, is obvious enough—and has been fully delineated in our review of Dr. Bretonneau's work, in our last number; but the rationale of the slow breathing is far from easy, nor are we satisfied with the explanation which the talented author has attempted, namely, the secretion from the ulcer in the throat of a morbid poison, "which may act by paralyzing or otherwise influencing the par vagum, or the branches of the eighth pair of nerves." In the fatal cases of cynanche maligna, where croup does not precede death, our author thinks it probable that the sinking of the living powers may be owing to absorption of the matter of the ulcer in the throat. But admitting this, why does not the slow breathing take place?

Be this as it may, Dr. Hamilton has adduced some curious and interesting cases, with the view of illustrating the subject on which he speculates. His attention was first drawn to the absorption of morbid poisons by cases of polypus uteri. In many such cases, there are symptoms of broken health, such as prostration of strength and œdematous swellings, although there may have been no hæmorrhage, but merely a trifling ichorous discharge per vaginam. On the removal of the polypus the discharge has generally ceased, and the health become restored. Our author was at a loss to account for these circumstances, but at last a case occurred, where the medical attendants were induced to delay the operation, and where the patient suddenly sunk a few days after the consultation. All the viscera were sound, and the body would have conveyed the idea of the patient having been carried off by some accident in the midst of health. A small ulceration, however, was discovered surrounding the peduncle, by which the polypus was attached to the uterus, "and it was concluded that the matter of this ulceration had been absorbed, and had acted as an animal poison." When we look around the surgical wards of an hospital, and observe the variety of ulcers that discharge all kinds of matter—when we see ulcerations of the stomach and intestines continuing for years, and where their discharges must pass along with the chyme through the tract of the intestines;—and all these without any thing like proofs of the dangerous effects of absorption, we cannot but regard Dr. Hamilton's mode of accounting for the sudden death of his patient as any thing but satisfactory. The next case is still less so. A stout woman was brought into the Royal Infirmary, in consequence of the sudden protrusion of a double-headed polypous excrescence of the uterus. A ligature was applied, and the tumour dropped off in the course of four or five days. Forty-eight hours after this, peritoneal inflammation was developed, and she died in two days. On dissection, no connexion could be traced between the uterus (which, however, was thickened and enlarged) and the peritoneal inflammation. So then, the removal of a polypus by ligature, and a succeeding peritoneal inflammation, are to be considered as illustrations of the absorption of a morbid poison into the system! We do not deem it necessary to make any remark upon this case. Valeat quantum valere debet.

Several years afterwards another case occurred, of which the following are the particulars. An individual, aged 45, of delicate constitution, was observed to have a polypous excrescence of the uterus, and a ligature was applied. The tumour dropped off on the fourth day. The next day she took some castor oil, which, not operating, was followed by some infusion of senna. Griping and pain in the belly succeeded—and the medical attendant was summoned. Twenty ounces of blood were abstracted. "*Oppressed breathing, and great rapidity and feebleness of the pulse succeeded, and she sunk within a few hours.*" No dissection took place; but our author thinks that it is "highly probable" the death was occasioned by the absorption of a morbid poison. Since this is merely matter of opinion, we may venture to give ours—namely, that the disease, or rather *disorder*, was *intestinal irritation*—and that the abstraction of twenty ounces of blood from a delicate female, under such a condition, was far more likely to be the cause of death than the absorption of a morbid poison. A dose of laudanum and carbonate of ammonia would, in our humble opinion, have been a much more efficacious remedy than venesection.

Dr. Hamilton adduces the cases of colliquative diarrhœa which so often occur after the opening of large abscesses about the hip-joint. We do not deny that pus may be absorbed, in such cases; but the fact is, that any great local irritation will often be transferred to the bowels, and diarrhœa is generally found to close the scene in most diseases.

But we must hasten to a close. Dr. Hamilton, finding that neither the application of leeches, the exhibition of purgatives, the application of blisters, nor the use of topical stimulants, as gargles, nitrate of silver, or even scarifications, were at all beneficial in lessening the extent of the local inflammation, in the throat affection above-described, resolved to take the earliest opportunity of endeavouring to lessen the amount of the diseased secretion. An opportunity at length occurred. On the 27th June, 1826, he was sent for to visit a boy aged six years, labouring under a modification of sore throat, which appeared to be of singular character. This was the third child in the family that had become affected with the disease. The first was a girl, nine years old, now sinking. In ten days after her attack, a little brother, four years old, was seized with the same symptoms, on which cynanche trachealis supervened, and proved fatal. When the state of the third patient was examined, there was found a slough on each tonsil, with a slight swelling of the velum, but no fever. He had only complained the preceding morning, and active measures had been pursued, such as purgation and leeching. In order to lessen the discharge, the sulphate of quinine was ordered in as large doses as the little patient could bear; but the medicine could not be got down in sufficient quantities. The ulceration was increasing, and fever was stealing on. Eight grains of the superacetate of lead, with 40 drops of laudanum, were mixed with eight ounces of rose water, and half an ounce was given every three hours. A solution of the same substance was also employed as a gargle. A dose of castor oil was ordered every second morning: under this treatment the sloughs gradually contracted, and the swelling of the velum pendulum progressively decreased, in the course of which it was discovered that ulceration had extended over the pharynx behind the velum, and the cure was completed on the 16th July. The whole quantity of superacetate taken was twenty-four grains.

From the cases which have fallen under our author's notice, he is inclined to believe that the disease is infectious. Dr. Hamilton concludes with some observations on the internal use of superacetate of lead, against which there seems to be a most unfounded prejudice and dread in the northen part of the union. No such prejudice exists among the "Southrons."

After he had written the paper, Dr. Abercrombie put into Dr. H's hands some account of Bretonneau's work on *Diphthérite*, most likely from this Journal, for we do not see that any other has yet noticed it. Dr. H. insists that he could not be deceived respecting the existence of ulceration in the throat—a fact (if it be one) which is doubted by Bretonneau, as he thinks the sloughs (false membranes) are usually taken for ulcerations.

In conclusion, we beg to state that we have no objection to any part of Dr. Hamilton's paper but the theory of morbid poison absorbed into the system, and thus causing death in angina maligna, and the polypous cases alluded to. All that can be said, pro or con, is matter of opinion, and, therefore, we shall dwell no longer on the subject.

---

### 6. PROPENSITY TO HOMICIDE, INFANTICIDE, AND SUICIDE.

1. M. Barbier, a physician of Amiens, in a late sitting of the Academy of Medicine at Paris, related the case of a woman, 24 years of age, who, having lost a child 3 months old, became the mother of a second. Ever since the trial of Mad. Cornier, this woman has been tormented with the dreadful propensity to destroy this second infant. The propensity was at first feeble, but gradually augmented, and, about 15 days previous to the date of report, the sight of a knife rendered this propensity so irresistible that the unhappy mother was forced to cry out for assistance, lest she should embrue her hands in the blood of her child !* M. Barbier had this woman admitted into the hospital at Amiens, in order to narrowly watch the case. In respect to her moral faculties, there was nothing extraordinary, except this unhappy propensity. Her intellectual faculties were perfectly sane. As to her corporeal health, she complained of alternate pains in the *epigastrium* and head—the *former* was always present when the infanticidal desire came on. Leeches to the epigastrium and head alternately have been employed, but, at the date of report, no change for the better had taken place. We are disposed to think that a little starvation would remove this propensity, as we have little doubt that it arises from a disordered state of the gastric nerves.

The above case excited the narration of several others by the members present. M. Marc lately saw a female, 32 years of age, the mother of several children, who had an irrisistible desire to destroy her offspring. She appeared to have no other physical ailment than intestinal irritation from worms. She has been taken into the Charenton Hospital.

M. Bricheteau stated the case of a young woman, of amiable disposition, the mother of two children whom she had nursed, who, having gone to Vincennes for the purpose of spending a few weeks of the summer, was shewn the spot where Papavione executed her criminal act. The sight of this spot made such an impression on her imagination that she has, ever since, been harrassed with the horrible propensity to murder her mother and one of her children ! Happily she disclosed the dreadful wish that lurked in her breast—she was removed from the place, and narrowly watched. By degrees the murderous desire subsided; but it was some time before she could bear the sight of her mother.

M. Esquirol stated that, since the trial of Madame Cornier, he has become acquainted with six instances of a parallel nature. Among these was a protestant minister, who became affected with the desire of destroying a favourite child. He struggled against this terrible inclination for 15 days,

---

* This may seem strange, or even contradictory; but instances of a similar nature are by no means rare. We are attending a gentleman, at this time, who has occasionally such a propensity to leap out of his bedroom window, that he orders his servants to tie him when he feels the desire coming on.

but was at last driven to the attempt on his child's life, in which he fortunately failed.

Vilermé related the case of a woman who, immediately after hearing the account of an assassination, was tormented, for three nights, with the desire to destroy her daughter, a girl 7 years of age. She had even secreted a weapon for the execution of her horrible purpose!

M. Bally observed that these cases have happened in all periods, nor did he believe that they were more common now than in former times; but only that they were more noticed. This does not appear clear to us, for reasons which will be stated presently.

M. Barbier asked the Society if any of them had seen such cases without some physical ailment? For his own part, he firmly believed that corporeal disorder was an indispensible preliminary condition—or rather predisposition. He knows a lady who is occasionally affected with monomania —"*but only when her stomach is disordered*"—" qui n'a cette monomanie que depuis qu'elle souffre de l'estomac."

M. Esquirol was quite of this opinion. All those whom he has seen affected with monomania were melancholics, and subject to stomach disorders.

M. Costel observed that all these facts, singular as they might appear, were explicable by a change in the sensibility—in short, on the principle of *morbid sensibility*.* In this condition, *example* has a most powerful influence. In illustration of this he mentioned the remarkable fact that, at the Hôtel des Invalids, a soldier having hanged himself on a post, his example was followed, in a very short time, by twelve other invalids—and that by removing this fatal post, the suicidal epidemic was put an end to.

M. Marc, in conclusion, descanted on the bad effects of giving publicity to the acts of suicide, infanticide, and homicide, now daily and hourly meeting the eyes of people in a nervous or melancholic condition, and leading to a multiplication of those acts themselves.

We have no doubt that this is one cause of the more frequent occurrence of these catastrophes now than formerly. But we believe that, in the progress of civilization, as knowledge is increased, and the processes by which it is acquired multiplied, so will be the causes of suicide and infanticide, but especially of the *former*. The mind will not be worked in disproportion to the body, without injury to the nervous system, and consequent injury to the body generally. This is the condition into which high civilization is leading man. It may have its advantages. That is quite another consideration:—but these are some of its evils. We believe, therefore, contrary to M. Bally, that the accidents above-detailed are on the increase among us in the present generation.

### 7. HYOSCYAMUS AND CAMPHOR.

Some further communications have been made to the Ed. Journal of Medical Science, respecting the above combination in affections of the bladder and urethra, by Mr. B. Bell. Camphor has long been known to have a peculiarly sedative effect on the urinary organs, and hyoscyamus is every day coming more and more into use, as a substitute for opium, in almost every kind of irritation and irritability. Mr. Bell remarks that

* Dr. Johnson has investigated this point at some length in his Essay on "Morbid Sensibility of the Stomach and Bowels."

camphor must be given in large doses, and frequently repeated—nor can we be assured that the system is under its influence, until the pulse be more or less affected—or even a slight degree of vertigo produced. Mr. Bell has found it necessary, in a few cases, to continue the employment of camphor, where the chordée, ardor urinæ, and irritable bladder, were in a high degree, even after the vertigo had taken place. But such is only necessary in extreme cases. Camphor, when given alone, is very apt to occasion nausea, heart-burn, and sometimes tremors. An anodyne, therefore, which does not occasion constipation, is a necessary adjunct. Hyoscyamus has been found to answer the purpose well, especially in the form of extract. The following are the formulæ which Mr. Bell generally uses.

1. R. Gum. camphoræ, gr. iij.
   Ext. hyoscyami, gr. ij. Misce ft pilula.
2. R. Gum. camphoræ,
   Ext. hyoscyami, aa gr. ij.
   Pulv. capsici, gr. i. Ft pilula.

No. 3 is a grain of ipecacuan instead of the capsicum. In general we find that Mr. Bell gives the above quantity, every third, fourth, or 6th hour, according to circumstances. The object is, to allay local irritation, without occasioning constitutional disturbance. In many instances the discharge of gonorrhœa ceases during the exhibition of the medicine, but this is not the object of the remedy—that object being to allay the distressing ardor urinæ and chordée.

---

### 8. CHERRY-STONE IN THE BRONCHIA.

Dr. Webster has related a case of this kind in the 5th No. of the *Med. and Phys. Journal.* A lad, 14 years of age, swallowed a broken cherry-stone, which slipped into the trachea, and produced a violent fit of coughing, with dyspnœa, and sense of suffocation. Twelve days after the accident he came under Dr. W's care, the above-mentioned symptoms still continuing without intermission, and with the addition of pain under the sternum. The stethoscope shewed the foreign body in the left bronchium, at the bifurcation of the trachea, thereby almost entirely obstructing the air from passing into the left lung. "On further investigation, it was also ascertained that the lung was sound, *though collapsed.* By percussion on the left side of the chest, a *deaf sound* was perceived, but in the right cavity every thing seemed healthy."* The patient had only expectorated some frothy mucus since the accident—pulse 120, small and sharp—great thirst—cough violent. Leeches, blister, antimonials—low diet,—absolute repose. He was relieved by these means. On the 16th day he was again bled, blistered, and the same medicines continued. These measures were repeated afterwards. From this period till the 68th day, the symptoms varied but little, and he was reduced to a state of extreme exhaustion. On the 30th day, it was ascertained by the stethoscope, that the air permeated more freely the left lung, particularly the upper portion. On the 68th day above-mentioned, he coughed up a large quantity of fetid pus, in which was found the cherry-stone. From this

* There must surely be some mistake here. If the lung was collapsed, air must have supplied its place, or water. In the former case, the sound would have been the reverse of *deaf.* We should say that the lung was *gorged,* not collapsed. This state would explain the phenomena. How was the cherry-stone discovered by the stethoscope? We apprehend that Laennec himself would not have been able to detect such a small body in the lungs.

time the patient gradually recovered, and was convalescent by the ninetieth day.

Dr. Gregory, Mr. Lawrence, Mr. Wardrop, Mr. Bacot and others, decided that *tracheotomy* should not be performed, unless the symptoms of suffocation became very urgent. We are at a loss to know what *tracheotomy* would have done for a case where the body was lodged in one of the bronchia. *Bronchotomy,* in the strict sense of the word, could alone have given the means of removing the cherry-stone, and this must have been done by opening the chest. He would have been a bold operator indeed who would have undertaken such a step! The remedy would probably have been at least as bad as the disease.

---

9. TREATMENT OF INTERMITTENT.*

Agues are not now nearly so prevalent in the fenny parts of Lincoln and Essex as formerly. The drainage of the lands has no doubt been the main cause of the amelioration; but, still ague prevails, and this is owing, Dr. Brown thinks, "to the mixture of salt and fresh water, an ascertained source of infectious miasmata." This, he informs us, arises from two evident causes—soakage, and the influx of the sea at the flowing tide into fresh water channels. The prevalence of intermittents and remittents during the last summer have furnished Dr. Brown with materials for the present Essay, of which we shall lay some of the particulars before our readers.

The principal object of the Essay, as the title imports, is the art of *repelling* the paroxysm. For this purpose, many means, medicinal, mental, and even mechanical, have been employed, as brandy, laudanum, emetics—ligatures, frictions, exercise—amulets and charms. All these have been occasionally successful, but few or none of them are now in general use. The interval between the paroxysms is almost universally waited for, in order to exhibit tonics—more especially bark or arsenic for the *prevention* of the succeeding attack. "The practice," says Dr. Brown, "of waiting for the paroxysm, and exercising powers to arrest it in its progress, on the first approach, has never been established." Dr. B. has known an ague resist the sulphate of quinine, with great obstinacy, for several weeks, and an example of this kind has induced our author to participate in the fears of Dr. Paris, "that evil not good would be the result of the new remedies." In this fear we do not participate; for, if any evil is to arise from arresting the progress of an ague, it is far more likely to result from clogging the stomach with a quarter of a pound of the ligneous part of bark, than from the exhibition of twenty or thirty grains of the sulphate of quinine.

Dr. B. does not come forward, however, to depreciate this last medicine, but with the view of "altering the manner of its exhibition, and promoting its operation by the assistance of others, by which its action becomes more uniform and certain." In March, 1824, Dr. B. was himself the subject of an intermittent, of tertian character, and with paroxysms unusually severe. He had been taking the sulphate of quinine, but still he had three attacks, and he looked forward with anxiety to arrest the fourth. For this purpose, he determined to alter his plan. Having taken a laxative medicine, and the attack shewing indications of approach, at one o'clock, the usual period, he commenced, by taking only two grains of the quinine, intending to

---

* A Treatise on repelling the Paroxysm of Intermittent Fevers; illustrated with Cases. By J. Brown, M. D. Boston, 1826, pp. 60. Underwoods' 1826.

follow it up with a much larger dose antecedent to the rigor, if the above quantity were not sufficient "But, in a very few minutes, its effects were apparent. The shrinking of the surface, the dejection of spirits, the painful sensibility to cold, head-ache, and thirst, and all unpleasant feelings, progressively subsided." From a dose so small, such influence over symptoms so formidable could hardly have been expected; and a temporary disappointment did succeed. In half an hour the attack returned, and again the same dose was repeated.

"Its influence over the rising symptoms became sensibly felt in a few minutes—the pulse became more full and distinct,—the respiration easy,—the cutis recovered its natural temperature, and, in eleven minutes, not one unpleasant feeling remained. This state, like that produced by the first dose, continued but a short time; in thirty-five minutes, the symptoms returned again in the same order as before, and a third time I repeated the quinine, in a dose of two grains; and now felt some confidence in its powers; and a hope that, by a steady perseverance in exercising this ascendancy I had gained over the approaching paroxysm, I should continue to repel, and ultimately to subdue it altogether. This was effected after the fifth dose, between which and the fourth, there had been an interval of an hour and a half, leaving a slight glow and moisture on the surface." 21.

Our author now proceeds to the narration of 32 cases of intermittent cured in the above manner, with more or less celerity. These we need not detail. A single case, the first on the list, will be a sufficient specimen of the practice pursued.

"*Case* 1. S. T. aged 17 years. This patient came to me the day following the fifth paroxysm. The ague was of the tertian type. She had been subject to habitual constipation of the bowels, and, under her present affection, it had become of more serious inconvenience to her. I therefore prescribed an active purgative to be taken at intervals, until the bowels were well acted upon, by which she got rid of several dark and offensive dejections.

"At the approach of the fit, she took the sulphate of quinine, beginning with the first dose as soon as she perceived the paroxysm coming on: an hour had elapsed before it became necessary to take another; and, at the expiration of a little longer interval than the first, she took the third and last:—the fit subsided, and the ague altogether left her. She took in all only six grains of the quinine." 22.

Active purgation appears to have been an indispensable preliminary to the mode of treatment; for, in one or two cases where this was omitted, the plan failed.

Dr. Brown's present opinion respecting the quinine forms a singular contrast with that which he has quoted from Dr. Paris, and which we have noticed at the beginning of this article.

"If any drug, now in use, is entitled to be considered *as a remedy fit only for kings*, the sulphate of quinine has a fair claim to the distinction." 60.

Upon the merits of the plan recommended and practised by Dr. Brown, we can pass no opinion, not having given it a trial. But we have now put our readers in possession of the facts, and they can easily test them in their own practice.

---

### 10. ENCEPHALIC PHLEBITIS.

The following curious case occurred in the practice of M. Gendrin and Dr. Leveque-Lasource, and is detailed in the Révue Medicale for April, 1826.

*Case.* Madame M———, aged 22 years, of good constitution, was safely delivered of her third child by Dr. Leveque-Lasource. She was in good health during pregnancy, but had a presentiment that she would not survive her accouchement. She did not suckle her infant. The doctor remarked that a little bouillie occasioned some febrile movement in the system. On the 7th day after delivery, while drawing her nipples with a glass, some blood issued, and frightened the patient very much. She had evinced, for a few days previously, but little regard for her infant. She fainted at the sight of the blood, and, on recovering, complained of cephalalgia of the left side. This pain was not diminished by two applications of leeches. On the 9th day, there was some difficulty of breathing, with pain in the side, which gave way to local bleeding. But the cephalalgia continued. The secretion of milk and of lochia had ceased. *10th day.* A new moral commotion, from the fire having caught the curtains. The head-ache still continued, in spite of leechings. *17th day.* Increase of head-ache, with pain in the left ear. Some symptoms of pneumonia requiring leeches. Sudden loss of power in the right arm, with numbness of the left leg. *18th day.* The paralytic symptoms are increased—the tongue is embarrassed. *19th day.* The paralysis augmenting—a large detraction of blood, local and general, which was cupped and buffed. This evening (1st Feb., 1826) M. Gendrin and M. Landré-Beauvais joined in consultation. The pupils were but little obedient to the light—tongue moist, but could not be pushed out—right arm completely insensible and motionless, but without rigidity—right lower extremity in nearly the same state—incontinence of urine—she appeared to comprehend, but could only say yes and no—sight and hearing undiminished—skin moist—pulse from 90 to 95, full and resisting. The patient indicated much suffering in the left side of the head. Blister—leeches to the groins. *2d. Feb.* A fit of great agitation came on to-day, with entire loss of speech, the pulse quickening to 136 pulsations, with some subsultus tendinum. Leeches to the head—cold applications—sinapisms to the extremities—twenty-four grains of calomel in six doses. *3d.* The symptoms evidently more favourable—paralysis diminished—pulse slower and softer—*same remedies as yesterday.*—*4th.* There were convulsive movements of the voluntary muscles, and other bad symptoms, which went on increasing till the 8th February, when death closed the scene. This event took place on the 26th day after accouchement, 17th from the commencement of the cephalalgia, and 9th day of the paralysis.

*Dissection.* This was not accomplished till after exhumation, on the fourth day from the patient's decease. A considerable degree of decomposition now obtained. On removing the skull-cap, it was observed that the middle cerebral vein of the right side was very much enlarged and tortuous as it approached the falx, where it opened into the superior longitudinal sinus. It was the size of a writing pen. They found it filled with a puriform substance of a yellow colour. In the tract of this vein the arachnoid membrane was slightly opake. The corresponding vein on the left side was in the same state. The whole of the superior longitudinal sinus itself was converted into a diseased mass which it would be difficult to describe, but which was infiltrated in many places with yellow purulent matter. There was organic disease to a vast extent in various other parts of the brain. Nothing remarkable in the other viscera of the body.

---

### 11. MR. CHEVALIER ON BELLADONNA.*

Mr. Bayley, of Harwich, drew the attention of the Profession to the em-

---

* Mr. Chevalier. Med. and Phys. Journal, No. 5.

ployment of belladonna, many years ago, in cases of tic douloureux, and other painful affections; and, more recently, Mr. Blackett has published some interesting papers on the external employment of this medicine.

Mr. Chevalier has now stated his experience of the efficacy of belladonna externally employed, in a number of complaints, all, however, agreeing in one particular—pain or morbid irritability. The first case in which he tried the belladonna was that of a stricture, attended with excessive irritability of the urethra. The extract, at first mixed with opium, and afterwards pure, was introduced on a bougie, and the morbid sensibility was soon reduced. To scrofulous glands, Mr. C. has applied the belladonna ointment, (equal parts of the extract and of some other ointment) with striking advantage. In many cases of inflamed periosteum, venereal nodes, scrofulous diseases of the bones and joints, the extract of belladonna has proved a soothing application. In one case where a tumour had formed on the back of the hand, as large as half an orange, and where the hand was condemned to amputation, the swelling dispersed in less than ten weeks, under the belladonna plaster first, and ultimately the pure extract. In the case of a boy, whose knee was so much diseased that amputation was recommended, but not submitted to—where the knee-joint was imperfectly anchylosed, the condyles enlarged, and the capsular ligament distended with fluid, the belladonna plaster (after the severity of the pyrexial symptoms was abated by proper local and general means) was employed, and, under its use, the joint diminished in size, the boy's health improved—and, by the use of splints and other mechanical means, the joint was brought nearly into a right line. In cutaneous affections, this remedy has proved very useful in our author's hands. Erysipelas, cancers, inflammatory and spasmodic affections of the chest, have all been benefited by the belladonna plaster. Tooth-ache, rheumatism, even of the acute kind, and many other painful affections, have been relieved by this narcotic application in the hands of Mr. Chevalier. In the cases of between two and three hundred persons, in private practice, and in the Westminster Dispensary, there were very few in which the remedy failed to do good—" none in which it appears to have done harm." This is very grateful intelligence to those afflicted with pain; but, like the account of all other favourite remedies, it must be taken *cum grano salis*. There can be no doubt that belladonna is a powerful sedative, and that it is more effectual than almost any other in reducing morbid sensibility of parts, and morbid contractility of the muscular fibre. It may, therefore, be more frequently used than it is, in cases of pain, irritation, and rigidity. On the Continent it has been much employed, especially by Madame La Chapelle, as an application to the os uteri, where that muscular ring continued too long contracted after labour pains had come on. In this country we believe it is seldom used for this purpose, and we have heard it objected to by an eminent accoucheur as dangerous, lest the paralyzing influence of the belladonna should extend farther than was intended, and render torpid the muscular fibres of the uterus itself. We know not whether this objection be well or ill founded.

---

### 12. ON COMPRESSION IN POISONED WOUNDS.

Dr. Bouillaud has been experimenting on the above subject, and has laid the results before the Academy of Médicine, and also before the public, in the September No. of the ARCHIVES GÉNÉRALES.

Dr. B. agrees with Dr. Barry, that we have no evidence of the agency of poisons through the medium of the nervous system, and that, if absorption can be prevented, the animal may be considered safe. The action of the cupping-glass is effected, of course, through the agency of pressure on the outside, and the removal of pressure on the inside of the glass; and Dr. B. was led to enquire whether simple pressure on a poisoned wound might not produce the same effects as the cupping-glass—and, also, whether a ligature applied above the wound might not have a similar effect. We shall state a few of the experiments made to determine these points.

*1st Ex.* At 14 minutes past 11 o'clock, he introduced three grains of strichnine into the cellular tissue of a rabbit's thigh, a ligature being placed above the wound. Seventeen minutes afterwards, the animal presented no symptom of poisoning; but at the expiration of 21 minutes, convulsions and cries manifested themselves. The thigh was then grasped very firmly with the hand at the place where the poison had been inserted, and soon the above symptoms of poisoning disappeared, and the rabbit tried to escape. During the 25 succeeding minutes, the manual pressure being continued, no sign of convulsions appeared. At 2 minutes after 12, the pressure was suspended, and, in six minutes afterwards, tetanic convulsions took place, which again ceased when the pressure was renewed, either by the hand or a cupping-glass strongly pressed on the part, without any exhaustion of the air. A slight diminution of the pressure of the glass was sufficient to renew the convulsions, and *vice versâ*. At 22 minutes after 12, the compression was definitively suspended; and, in order to spare the animal any farther sufferings, some Prussic acid was dropped into one of its eyes, and it quickly expired.

*Exp.* 2. At 22 minutes past 11, Dr. B. introduced three grains of strichnine into the thigh of an adult rabbit. In eight minutes, cries and convulsions, with rigidity of the members, manifested themselves. The part was then strongly grasped with the hand, and almost immediately the cries and convulsions ceased. The compression was suspended, and again the convulsions returned. A cupping-glass was then strongly pressed on the part, without exhaustion, and the symptoms of poisoning were quickly arrested. The cupping-glass was removed, the convulsions returned, and so on. Pressure with the hand had the same effect. The rabbit was set free, having first washed the wound, but it soon died. Nine experiments are detailed, but all with the same results. These results are, we confess, very curious, if they are faithfully related, which we have no reason to doubt. From them it would appear, that the best plan in cases of poisoning, say, for instance, the bite of a mad animal, would be, to apply a ligature as soon as possible above the wound, and also the exhausted cupping-glass to the wound. Leeches might also be applied in all directions below the ligature, by which means the poison would be extracted, and any blood which might happen to be contaminated by that poison.

Reflecting on the above experiments, we are at a loss to account for the manner in which simple pressure relieves the symptoms of poisoning when they have once occurred, and that, too, in so sudden a manner as is here represented. If the experiments be correct and faithful, it would really appear to us, that the explanation of this sudden relief is more easily furnished by the nervous, than by the vascular or absorbent systems. Strong pressure on the wound might deaden the sensibility of the nerves there, and thus take off, for a time, the local irritation of the strichnine, the cause of the convulsions. This, of course, is reasoning on the possibility of poisons acting through the medium of the nerves—and when we consider the rapidity with which some animal poisons act—as that of the cobra de capello, &c. we are almost irresistibly led to believe in the instrumentality of the nerves, on some occasions at least. We leave it to physiologists to determine whether the above experiments of M. Bouillaud be in favour of the nervous or vascular agency of poison introduced into a wound.

### 13. ABDOMINAL DROPSY, WITH PREGNANCY.*

We have frequently directed the attention of our brethren to this subject, and we believe we have collected more information on the complication in question than can be found in any other work. We add the particulars of the following case to those already on record.

Mrs. A. aged 40, robust, and the mother of three children, stated that she miscarried on the 15th Jan. 1825, being the third accident of this kind, and always at the end of the third month. Having caught cold at this period, she soon began to feel a fulness of the abdomen, which gradually increased. Though the catamenia continued regular, yet she supposed herself pregnant. On the 11th July, when she applied to Mr. K. she was the size of a woman in the 5th or 6th month of utero-gestation—countenance sallow—urine scanty—bowels costive—shooting pains in the left side—general health not affected. The abdomen became more and more distended, and, by the 1st October, it was necessary to draw off the water, when 27 pints were evacuated. In a short time she was able to do the duties of the house. Still she suffered pain in the left side, and the fluid began to collect in a few weeks afterwards. By the 30th January, 1826, she was as large as before. She now stated that she felt something like the motion of the child, but, from the anasarcous state of the abdomen, it was impossible for Mr. K. to determine the point. The lower extremities were much swelled, and one of them gave way. The catamenia had not returned since June.

On the 11th February, she was suddenly delivered of a small living child, of 7 months. After delivery, the abdomen was very little reduced in size. On the 21st Feb. she complained of general pain in the bowels, short breathing, cough, inability to lie on the left side. She now became affected with hiccup, high fever, black vomit, and all the symptoms of puerperal peritonitis. Yet she struggled through, and got so far recovered as to be able to move about. But the dropsical swelling increased, and, on the 19th of June, she was again tapped. Only one quart could be got to flow, from what cause was then unknown. After this, the patient's health gradually declined, and she died on the 29th June.

*Dissection.* Thirty-six quarts of water were drawn off by the trocar. On opening the abdomen, an immense sac appeared, covering completely all the viscera. It was easily detached by the fingers, except where it was connected with the broad ligament of the uterus. There it required the scalpel. A great quantity of shreddy matter adhered to the inside of the sac. The ovarium of the left side was enlarged, and enveloped in a mass of hydatids. The right ovarium was not so much enlarged, but equally enveloped in hydatids. The other viscera were healthy. It was now discovered that the trocar had not entered the sac in the last operation. There can be no doubt that the sac was an hydatid.

### 14. DISEASE OF THE BRAIN WITHOUT CORRESPONDING SYMPTOMS.

When we find disorganization of a part in the dead body, where few or no symptoms revealed its existence during life, we are always surprized; but when this part happens to be the brain, the sensorium commune, we are confounded in all our calculations.

A man was received into one of our public hospitals for an accident—a fracture, which got well, or nearly so, in due time, and when he was on the eve of departure, he suddenly expired in his bed, before any assistance could

* Mr. Kesson. Ed. Journal Med. Science, No. 4.

be offered to him. On dissection, all the viscera of the body were sound, except the brain. Here was unequivocal evidence of chronic inflammation of the arachnoid, that membrane being thickened and opake. The pia mater was infiltrated with serous effusion, and more than four ounces of water were found in the lateral ventricles. No symptoms during life indicated the existence or progress of such a disorganization; and we can only account for such a circumstance by the slowness of its march. Instances of this kind are recorded by many observers, and they teach us humility in our prognostic and diagnostic enunciations.

---

### 15. EXCISION OF THE HEAD OF THE HUMERUS.

This severe operation has been lately performed by Mr. Syme, of Edinburgh, in the case of a woman, who had a diseased shoulder-joint of several years' standing. The operator made a perpendicular incision from the acromion, through the middle of the deltoid, extending nearly to its insertion, and traversing an old sinus. On introducing his finger, he felt that the disease was almost entirely confined to the head of the humerus; he, therefore, formed, by a semilunar cut, a flap from the scapular portion of the deltoid—exposed the joint—insulated the head of the bone—protruded it—and sawed it off, without any injury to the soft parts. The glenoid cavity of the scapula was found quite sound, except that it was deprived of its cartilage. A portion of the acromion was bare and rough; it was, therefore, removed by the cutting plyers. There was a smart hæmorrhage at first; but the only artery of consequence which was divided was the posterior circumflex. This was compressed with the finger till the operation was finished, and then tied. The flap was now replaced, and retained in perfect apposition by five or six stitches of the interrupted suture. Simple dressings were applied—the whole operation occupying ten minutes. Some fever followed, and was met by proper antiphlogistic measures. Erysipelas and some degree of sinking required stimulants, and the cure went regularly on to a favourable termination. The limb is shortened about an inch, and the shoulder-joint is susceptible of distinct motion in all directions by means of its own muscles, while all the other joints of the limb are as serviceable as they ever were.

This bold operation is creditable to Mr. Syme, who has thus remedied a disease of the shoulder-joint that had existed for seven years, rendering the arm entirely useless—embittering the patient's life—and threatening to destroy it altogether. This operation, originally performed by Mr. White, of Manchester, and by one or two other surgeons, has, for a great many years, fallen into disuse. Perhaps Mr. Syme's case may excite the attention of surgeons to it once more.—*Ed. Journ. July*, 1826.

---

### 16. WANT OF PERICARDIUM.*

There are many cases on record where the pericardium is said to have been wanting; but, as most of these observations were made at a time when inflammations of the heart and its envelops were not much known, it is supposed that many of the cases related by authors, were those of adhesion between the free and reflected sheets of pericardium. This may be true; but still, to assert with Haller, that this error was always committed, is not in accordance with M. Breschet's experience. The following is one of M. Breschet's reasons for questioning the scepticism of Haller.

---

* Repertoire, No. 1. M. Breschet.

*Case* 1. A young man, 28 years of age, tall and thin, enjoying habitual good health, was received into the Hôtel Dieu on the 5th Dec. 1825, for a severe intestinal inflammation. He evinced no symptom of any affection of the chest, functional or structural. The abdominal inflammation resisted every measure, and the patient died three days after he was received into the hospital. The symptoms were those of acute dysentery.

*Dissection.* The coats of the intestines were found thickened and ulcerated. There was no trace of pericardium, the heart appearing free in the left side of the chest. The heart, lungs, and large vessels were carefully taken out, and laid before M. Breschet, who examined them with the greatest care, and avers that the pericardium was completely wanting. It was supported in its place entirely by the vessels which arose from it. The preparation was presented to the Philomathic Society, and examined by many anatomists, all of whom concurred in the statement of Breschet.

Our author cites several cases from former writers, and especially the case related by Dr. Baillie, in support of the opinion which he has given in opposition to Haller.

---

### 17. REMARKABLE CASES OF DYSPHAGIA.

1. An interesting case of this kind lately occurred in Guy's Hospital, the patient being a young woman from the country. Her difficulty of swallowing being very great, and yet the œsophagus not presenting any obstruction to the introduction of a bougie, the medical officers had some suspicion of imposture. The young woman had much cough and profuse expectoration, which, with the dysphagia produced rapid emaciation. The patient left the hospital without relief, and soon afterwards died in the country, being literally starved by the inability of swallowing food. The body was examined after death, and the morbid parts shewn by Dr. Burne to the Physical Society of Guy's Hospital, on Saturday night, the 21st October. There was a fistulous communication between the trachea (a little above its bifurcation) and the œsophagus, an inch in diameter, the edges being perfectly cicatrized. There was no disease, either in the trachea or œsophagus. The lungs were tuberculated, and purulent depôts existed there.

The great curiosity of this case is, the possibility of swallowing at all, without a portion, at least, of the aliment, whether solid or fluid, getting into the trachea at each attempt. For our own parts, we believe that some of the aliment did get into the air passage, since it was observed in the hospital, that every attempt to take food was accompanied or followed by great distress—generally by vomiting and coughing. It was argued in the Society, that Nature had made some effort to obviate this accident, in the same way as she does when the epiglottis is destroyed by ulceration, and where the power of swallowing still continues. But the cases are not analogous. The muscles about the rima glottidis have the power of shutting that aperture, when any thing else than air is presented; but this could not have been the case, where a breach was made between the trachea and œsophagus. Our conviction is, therefore, that the dysphagia arose from this very circumstance—namely, the escape of matters from the gullet to the air passage, causing the distress that was complained of. How long this unnatural aperture had existed, could not be ascertained, but we understood that the dysphagia had been complained of for some years. We should suppose that the aperture was caused by ulceration, and remained fistulous afterwards from the transit of matters through it.

2. Dysphagia, with Organic Disease.

In one of the late sittings of the Royal Academy of Medecine in Paris, M. Kerkaradec presented a curious case of organic disease of the pharynx, with dysphagia. A woman, 60 years of age, of melancholic disposition, was subject to a convulsive action of the pharyngeal and laryngeal muscles, on attempting to swallow food, which threatened suffocation. After spitting out precipitately the first morsels of food, she was then able to swallow pretty well. These attacks were at first separated by long intervals ; but these gradually decreased, and a sore throat supervening *rendered* deglutition of solids almost impracticable. When she did swallow, she was obliged to lie down in the horizontal position. Nothing could be seen in examining the throat, and the disease was considered to be spasmodic ; but the poor woman continued to waste away, while a considerable salivation was established by the habit of spitting out for fear of the attempt to swallow the saliva. To the dysphagia was now added a difficulty of inspiration, as if the breath was mechanically impeded. The inspiration was accompanied by a hissing noise. The region of the larynx, on the sides, became rather tumefied, and then the medical attendants began to fear they had something more than spasm to combat. Moxas were applied, and mercurial with iodine ointments were rubbed on the neck. These and various other remedies were inefficacious, and after four months of impending asphyxia, death kindly put an end to her sufferings.

The larynx was found healthy, except that it was rather narrower than usual at its inferior part. The internal membrane of the glottis was a little red and swelled, and at its upper and posterior part there were whitish vegetations, apparently springing from the pharynx. The pharynx itself was covered with vegetations, so that its lining membrane could not be distinguished. These vegetations had the cauliflower appearance. In the neck were a number of small encysted tumours containing a suetty substance. M. Kerkaradec considered the vegetations and these encysted tumours to be of the same nature, but varying in appearance, in consequence of the different textures in which they were developed. He asked if they were not all of a tuberculous nature ? No answer seems to have been given ; and the probability, we think, is, that the disease was tubercular.

The above was a case where much benefit would have been derived from a careful introduction of bougies, and a regular application of the solution of nitrate of silver, proportioned in strength to the varying susceptibility of the parts. We wonder that these means were not tried for the relief of the unfortunate patient.

Two other cases of perforation of the œsophagus and stomach were read in the same Society, by M. Léveillé.

*Case* 1. A man, æt. 22, was bitten in the right hand by a dog which he *thought* mad. The wound healed, but six weeks after the bite, he was suddenly seized with violent pain in the right shoulder and side of the chest ; sense of suffocation, difficult deglutition, vomiting, delirium and death in the course of the night. On dissection the œsophagus was found perforated, with extravasation into the posterior mediastinum.

*Case* 2. A man, æt. 55, suffered for a year from occasional syncope, succeeded by constipation and symptoms of disease of the stomach, without, however, vomiting or apparent tumour. Violent pain in the hypochondrium came on, one night ; belly distended—death. *Dissection.* An oval opening on the anterior surface of the stomach, near the pylorus. On the borders of this perforation were several yellow tubercles about the size of barley-corns, and the contiguous mucous membrane was destroyed. The left auricle of the heart was double its natural size, and from the septum, between it and the right, grew a medullary tumour, forty-five lines in circumference.

### 18. DIABETES MELLITUS.

Dr. Bardsley, of Manchester, has lately detailed, with great minuteness, a rather interesting case of this disease. The patient was a weaver, 38 years of age, who was admitted into the infirmary making at the rate of 19 pints of sweet urine in the 24 hours. It was attended with the usual symptoms—dry skin, voracious appetite, thirst, pain and weakness in the loins, spongy ulcerated gums, constipated bowels, &c. He was put upon a strictly animal food diet, with drink containing the nitric acid. Leeches were also applied to the loins. By this plan the symptoms were much relieved, and the urine diminished to five or six pints per diem. He thought himself so well that he requested to leave the hospital. In the course of a few months he relapsed, not being able to procure a sufficient supply of animal food. The bleeding plan was then put in force, and a fair trial given to it, but it completely failed. Then Dr. Bardsley had recourse to opium, a grain of which was given three times a day, the diet being the usual run of the house. This also failed. The original plan of animal food diet was accordingly resumed, and steadily persevered in, till the urine was reduced to five or six pints, with very little saccharine quality, and he was ultimately discharged, apparently cured. He returned several times afterwards to make report of his continuance of health.

As far as one case goes, this affords satisfactory evidence of the superiority of the animal food regimen over the other plans of treatment mentioned above. Where there is no unsound organ, as the lungs, we shall occasionally succeed in curing diabetes; but unfortunately it does not often happen that such uncomplicated cases present themselves to our notice.—*Ed. Journ. Med. Science.*

### 19. CLINICAL OBSERVATIONS. BY DR. BLAND.*

*Case* 1. *Cerebral Hæmorrhage.* On the 29th August, at 7 o'clock in the morning, Jean D——, aged 68 years, was found in the court-yard of his residence stretched out, with his head lower than the rest of his body, being insensible and motionless, his face puffed and livid. He was instantly carried in, and a few minutes afterwards he revived, but with hemiplegia of the left side. When Dr. B. visited him half an hour after this, the facial tumefaction had subsided—the left side of the lips was a little depressed—the tongue inclined to that side—the upper eye-lid, on the left side, drooped, and could not be raised, and that eye was rather dull. He pronounced with difficulty, yet the intellectual functions appeared free. The left side was paralyzed—the upper more than the lower extremity. The natural sensibility of the paralyzed parts was unaffected. The pulse was 90 in the minute, and full—the heat natural.

The patient reported that, having been very much constipated, he had strained violently at stool, when he was seized with vertigo, and was unconscious of what occurred subsequently. *Bled to 12 ounces, and 20 leeches applied behind the right ear.* In the evening, the tongue was more free—the lower extremity less paralyzed, and even the upper extremity; beginning to

* Bibliotheque Medicale, Septembre, 1826.

have some slight degree of muscular power in the fingers. This improvement gradually increased, and, by the 17th September, there was no paralysis left.

*Reflexions.* On a former occasion, Dr. Bland had published some observations on the influence of great muscular exertions in the production of apoplexy; and considers the above as a case in illustration. It requires but the effort to strain while the breath is held in, to convince any one that blood is thereby accumulated about the head. If predisposition to apoplexy obtains at the time, such as weakness, or disease of any of the cerebral vessels, there can be no doubt that this straining may be the immediate cause of extravasation on the brain. That the extravasation was to a very small amount in the foregoing case, we think is evident, from the rapidity and completeness of the cure. But that there *was* extravasation, we have not the smallest doubt.

*Case* 2. *Pharyngitis, with acute fever.* A female, 35 years of age, after exposure to wet and cold, on the banks of the Rhone, was seized with general malaise, chills, and heats. Two days afterwards, anorexia, pains in the limbs, fever, acute pain in the pharynx, with great difficulty of swallowing. In the same evening the fever was intense, with head-ache, &c. *Barley water. Third day.* The symptoms still more evident—pulse strong and full —difficulty of swallowing increased. *Barley water and warm pediluvia.* The patient fainted away while the feet were in the water, and this syncope continued for the space of 15 minutes. At length the patient revived; but the fever and pharyngitis were gone, to return no more.

Dr. Bland occupies many pages with reflections on this case, and in attempts to prove that syncope has a tendency to check inflammation. In this country the principle has long been known, and syncope is often aimed at in the treatment of severe local inflammation. But in France, where blood-letting is hardly ever carried to such an extent, the effects of syncope are but little understood. The practice pursued by Dr. Bland, in the above case of severe pharyngitis, is a specimen of the Hippocratic practice still prevailing in that country.

### 20. REMARKABLE CASE, BY C. MULLER.*

*Case.* A corporal in the Sleidgwick Cuirassiers, while assisting a man in putting up hay into a loft, fell back on the ground, and on being raised, it was discovered that he had lost the power of his upper and lower extremities. Surgeon Muller was called to his assistance in half an hour after the accident, and found the soldier pale, but sensible and powerless. The circulation and respiration were unaffected; but the lower half of the body was dead. Feeling was extinct in all parts of the body. There was no external wound; but the man had evidently fallen with his back across a cylindrical block of wood. M. Muller could not ascertain whether or not the spine was luxated. Blood-letting was practised, and a purgative exhibited. In the night, the patient was agitated and delirious. Next morning, he talked rationally, and he was more animated in his countenance; but the pulse was feeble, and he felt convinced that he was dying. He died a few hours afterwards.

On examining the spine, blood was found extravasated between the muscles and the vertebræ, as also into the theca vertebralis, from the sixth cervical to the ninth dorsal vertebra. The brain was perfectly sound, as were all the other viscera of the body.

* Biblioth. for Lœger, 1824.

### 21. CASES OF SOFTENING AND PERFORATION OF THE STOMACH IN CHILDREN. BY DR. KRUGER.

*Case* 1. This was an infant consigned to an ill-tempered nurse, who had moreover some suspicious copper-coloured spots on her body. When Dr. K. saw the child, it was then six months old. It was peevish, and evidently laboured under disorder of the primæ viæ, as evinced by acid eructations, vomitings, and green stools. The doctor prescribed alkaline absorbents, tonics, and nourishing diet, with warm baths, and good air. There was a slight amelioration of the symptoms. But they returned from time to time, and were greatly exasperated by the application of a sinapism to the epigastrium, soon after which, the infant expired in great torture.

*On dissection*, the stomach and duodenum were found much distended with gas; and a very gentle movement of this last intestine, for the purpose of separating it from the stomach, caused it to burst. Its coats were softened along the whole exent of the great curvature, into a kind of bouillie, and the mucous membrane was of a brown or greenish colour. In the small intestines there were seven or eight invaginations.

*Case* 2. This was a little girl, three years of age, who had been confided, like the other, to a nurse suspected of being syphilitic, and who had exhibited copper-coloured spots on the skin. Her food having been of an indigestible nature, and bad in quality, she began to shew symptoms of gastric irritation, and diminution of strength. M. Kruger exhibited mercurial medicines, alternated with tonics, and, by these means, the health received a temporary improvement. Suddenly, however, the following symptoms were developed:—general coldness—tumefaction of the limbs—slow respiration. Next morning these symptoms were changed to vomiting and diarrhœa. The irritation ultimately spread to the chest and to the head, and convulsions closed the scene. Stimulants had been administered in this case, both internally and externally.

*Dissection.* Slight redness of the air-passages—effusion in the chest, and also into the abdomen. The stomach was of a brownish colour internally, and its coats so softened for a considerable space, that they went into a kind of mucilage between the fingers. This disorganization was chiefly found in the great cul de sac of the stomach, and gradually disappeared towards the superior part of the organ. The fluid found in the stomach was slightly acid, as in the former case.

These cases are analogous to some that have been recently published in this country—especially by Dr. Gairdner, and are not devoid of interest.

We shall here state a case of perforation in an adult, which happened in this country.

*Case.* 3. This is related by Mr. Syme, in the October number of the Old Edinburgh Journal, of which the following are the particulars.

J. T. aged 23, had been dyspeptic from childhood, and when he applied to Mr. S. he complained of pain in his stomach, very severe after meals—habitual costiveness—dark-coloured stools—tenderness in the right side of the epigastrium on pressure, without tension there. He was put upon a system of diet, and ordered pills composed of equal parts of the mercurial and compound rhubarb masses. About a month after this Mr. S. saw the patient, who had experienced no relief, and was emaciating. On the 1st. of July, 1825, he dined more fully than usual, and in the evening, was observed by his workmen to put his hand frequently on his stomach. He remarked that he thought he could not live long. In the night he was seized with severe pain in the epigastrium, which he considered to be cramp, and employed hot fomentations, &c. These proved unavailing, and Mr. S. was called in. He had now severe pain in the stomach and unquenchable thirst, with retching and vomiting. The pain was relieved by pressure—pulse neither sharp nor much accelerated—tongue clean and moist. Bled to 16 ounces—a full dose of opium—sinapism to the pit of the stomach. In the morning, no relief, except of vomiting. A warm bath, and a dose of castor oil, followed by an enema. The oil did not operate, and the enema returned without fæculence. Pulse was now 124 and sharp—pain more extended—abdomen tense and tender. Bled to 30 ounces—salts and senna. July 3d. All the symptoms aggravated. At 4 o'clock he died.

*Dissection.* Abdomen tympanitic—some air and muddy fluid in the peritoneal cavity. The peritoneum itself was of a brownish red colour, from great vascularity—stomach externally of its natural colour, but much distended with air. On pressure being applied to this organ, air escaped through its parietes. On opening the stomach there was found an aperture, the size of a sixpence, in its lesser curvature, near the pylorus. The margins of this hole were regular, white, and firm, resembling ligament, being somewhat thickened. There were neither ulcers, cicatrices, nor inflammation in any other part of the mucous membrane. Some of the contents of the stomach had escaped through this opening into the abdomen.

*Remarks.* There can be no doubt that this aperture was an ulcer of long standing, in the stomach. The case is very nearly similar to that which we related some time ago, as happening to Mr. Barber of Covent Garden. In both cases, the rupture of the bottom of the ulcer took place after imprudent repletion. It is highly probable that ulcerations of this kind would occasionally heal, if the patient could be persuaded to adopt a most rigid system of diet on farinaceous food. But there are few who will forego the luxuries of the table, even when warned of the dangerous predicament in which they stand!

*Case* 4. In a late sitting of the Academy of Medicine, M. Leveillé read a report of two cases of spontaneous perforation of the stomach, of which we shall only notice one. A man, 55 years of age, had experienced four attacks of syncope in one year, and afterwards symptoms of derangement of the stomach, but without vomiting, tumour, or constipation. At length, there came on suddenly in the night, acute pain in the hypochondrium, distention of the abdomen, &c. and death took place before the morning. On dissection, there was found an oval hole on the anterior surface of the stomach, of small diameter, near the lesser curvature, three or four inches from the pylorus. Around the perforation were several minute yellowish tubercles. The mucous membrane was eroded for some space around the perforation. The left auricle of the heart was double its natural size, and to the mitral valve was attached an encephaloid tumour, forty-five lines in circumference—the probable cause of the attacks of syncope.

---

### 22. EPILEPSY CURED BY NITRATE OF SILVER.

Dr. Balardini has added one to the many instances on record of the efficacy of this remedy in epileptic fits. The patient was a young woman, aged 21 years, who, from her infancy, was subject to this complaint. It had now become very severe, very frequent, and the paroxysms often followed by temporary alienation of mind. Various remedies had been used in vain, when Dr. B. gave the argentum nitratum a fair trial. He commenced with two grains a day, and gradually increased the dose. The medicine was continued three months, and never produced any bad effects. Occasionally it acted slightly on the bowels. The patient was cured.

We are inclined to think that this medicine will be more used than heretofore. If epilepsy be dependent on organic disease of the brain, we do not see that the nitrate of silver can do any harm, but probably good, if due attention be paid to local evacuations from the head, and counter-irritation. If, as is very often the case, the disease be connected with irritation in the primæ viæ, there is no other medicine more likely to remove this irritation, or at least to remove the morbid sensibility of the gastric and intestinal nerves, than the nitrate of silver.

### 23. ERGOT OF RYE.

In some recent Italian Journals we find several cases related where this medicine exhibited its specific effects on the uterus in a very unequivocal manner. In an animated discussion in one of our metropolitan medical societies, this season, an eminent accoucheur contended that the ergot of rye could hardly ever be required for the expulsion of the fœtus, since the efforts of Nature would always, if time were granted her, bring the child within reach of the forceps, when it could be extracted without danger or difficulty. He also contended that if the os uteri were not dilated, or, at all events, not easily dilatable, the violent contraction of the uterus, occasioned by the ergot, might be productive of dangerous effects, and probably rupture the organ. It was contended, on the other hand, by some able obstetric practitioners, that, where the os uteri was dilated or dilatable, there could be no just reason for waiting days together for the slow operation of Nature, and that, in such cases, the administration of the medicine was proper and justifiable. Several cases were related by some of the members present, where the medicine was administered with advantage, and without any bad consequences. Upon the whole, we are inclined to agree with the last class of practitioners, and we think that, under the restrictions above-mentioned, respecting the state of the os uteri, the ergot of rye may occasionally prove a useful auxiliary to the languid powers of the uterus.

### 24. AN OVER-DOSE OF HYOSCIAMUS.*

The widow Rankin and her daughter had breakfasted on what they supposed to be hyssop tea, but which, in reality, contained an almost entire young plant of hyosciamus niger. The mother, who had taken a larger dose than the daughter, was soon seized with giddiness, vertigo, &c. and when Mr. D. arrived, he found the old woman in a very merry mood, being completely delirious, singing, and imitating with her hands the occupation of spinning. Her pupils were dilated—hands and feet cold—face pale, and covered with perspiration—pulse small and irregular—and she was incapable of comprehending what was said to her. The daughter was not nearly so much affected. Six grains of tartar-emetic, and a repetition of the dose, dislodged the enemy from the daughter's stomach; but it required eighteen grains of tartar-emetic, two scruples of sulphate of zinc, one scruple of sulphate of copper, and three drachms of ipecacuan powder, before the contents of the old woman's stomach could be cleared away. She soon got well, and felt no inconvenience from the strong doses of emetics. Mr. D. regrets not having a stomach-pump, or any thing that would form a substitute. We

* Case of poisoning by Hyssop tea. Mr. Donaldson, of Stonehaven. Ed. Journ. Med. Science, No. 4.

would strongly advise the surgeons of a town to have at least one stomach-pump *pro bono publico,* which should be kept in a place where any one of them could have ready access to it in cases of emergency. The expense would be trifling in this way—and some lives, as well as reputations, would probably be saved by this small JOINT-STOCK-COMPANY.

### 25. SULPHATE OF QUININE IN FRICTION.

A memoir was lately read in the Parisian Academy of Medicine, by M. Pointe, a physician at Lyons, on the use of sulphate of quinine, employed in the way of friction on the gums and inside of the mouth, in fevers of the intermittent and remittent type. M. Pointe, being apparently a disciple of Broussais, had the fear of "gastro-enterite" in his mind, and found, or fancied he found, the stomach irritated by the internal use of the quinine. He, therefore, used it in the above manner, and the fevers were cured. A lively discussion ensued among the different partisans, and some of them stoutly denied that there was any gastritis or enteritis in these cases; but only an "etat bilieux," for the relief of which emetics ought to have been prescribed, and then the quinine would have succeeded better. The exhibition of emetics, however, was against the Broussaian doctrine, and was much condemned. But if "*gastro-enterite*" be the proximate cause of these intermittent and remittent fevers, we much wonder how the quinine should have cured the disease, in whatever way it was administered. We believe, with M. Miguel, the reporter on this memoir, that this same quinine does not act merely as a *revulsive,* the sophism of Broussais, but "*by a specific, though unknown operation on the stomach.*"

### 26. BRONCHOCELE.

M. Angelot, of Grenoble, has transmitted a memoir to the Royal Academy of Medicine, of Paris, on goitre. This curious disease, endemic among the Alps, affects a great number of the soldiers sent there from other countries. They generally begin to shew symptoms of bronchocele in three or four months' sojourn among the mountains; but all these goitres are readily cured by the external use of iodine ointment. M. Leveillé observed that, in many instances, it was only necessary to remove from the goitrous country, to be cured of the disease, and cited the case of two persons affected with goitre, by some residence in Switzerland, and who got rid of their complaints by a removal to Paris. M. Desgenettes wondered that the author did not notice one of the principal causes of goitre, the snow-water used by the soldiers. He had been able to preserve whole regiments from bronchocele, by prohibiting this water, and causing the men to bring pure water from a spring for their daily use. But here we think Desgenettes has made a great mistake. That *snow-water* has little or nothing to do with the production of goitre is proved both in Switzerland and other countries, by attention to localities. How is it that bronchocele is so prevalent in Sussex, and so rare in Scotland? The reverse should obtain if snow-water were the cause of the disease. Again, how is it that bronchocele is scarcely seen in the valley of Chamouni, where the water is nothing else than drippings from Mont Blanc—while on the other side of the Col de Balme, in the Valley of the Rhone, we see hardly any thing else than bronchocele and cretinism? That the cause is in the water, we have no doubt; but the snow does not appear to be connected with the etiology of the disease. M. Desgenettes may have preserved his soldiers from goitre, by causing them to drink from a spring; since it is highly probable that the river-waters of Switzerland contain the deleterious agent, whatever that may be. It is fortunate, however, that, in iodine, we now possess a very powerful counteractor of this mysterious poison.

---

### 27. BROUSSAIS AND CLUTTERBUCK IN DANGER.

The fevers which have prevailed during the summer and autumn in London, shewed such a low type, and affected so many different organs, that even the disciples of Dr. Clutterbuck have maintained, under his own presidency, in Bolt-court, that the mucous membrane of the stomach and bowels was much more frequently the primary seat of fever than the brain. This is a melancholy circumstance. No theory of fever is now likely to last more than a few years; for these vile *constitutions* of the elements around us mock the sublimest genius, and turn all his fine-spun hypotheses into ridicule!

In Paris, dismay prevails among the "gastro-enterite" gentry. M. Honoré has had the courage to proclaim before the Academy of Medicine, that the reigning fevers, as well as other diseases of the French Metropolis, have changed their character; and that, instead of being inflammatory (as they have been for 20 years past) and requiring bleeding and antiphlogistic treatment, they are, on the contrary, exasperated by these means, and require the quinine! In the hospital Necker, as well as in private practice, he has had numerous cases under his care, where, "notwithstanding the symptoms of gastric irritation, they were increased by bleeding and leeching, and cured by bark." The phalanx of Broussais made, of course, a decided stand against this rebellion, and overpowered by numbers the anti-gastric party. But even the disciples of Broussais were forced to acknowledge that quinine did cure periodical fevers—"même de Nature inflammatoire;" but then they thought it was only when the gastro-enterite had begun to subside! What shifts and subterfuges will the theorists fly to, when facts fail them! The editors of the ARCHIVES wisely remark, that M. Honoré's paper was very useful as a check on the abuse of antiphlogistics in the reigning fevers. "We must not," say they, "confound fever with inflammation, nor conclude that all fevers have local inflammations for their bases." They maintain that there may be *inflammations as well as fevers of an asthenic* nature, or at least arising from asthenic causes, and consequently not bearing antiphlogistic treatment.

---

### 28. INCONTINENCE OF URINE.

M. Samuel Lair read lately to the Academy of Medicine a memoir on this subject. He refers most cases of incontinence of urine to a want of equilibrium in power between the body of the bladder and its neck—the latter being in an atonic state. The failure of tonics, in these cases, he attributes to their acting equally on all parts of the bladder. He therefore imagined that, if he could stimulate the neck of the bladder and not the body, he might have better success. With this view, he introduced, by means of a catheter, tincture of cantharides so as to touch the urethra in its prostatic part, and also the neck of the bladder. By this process he cured three patients who laboured under incontinence of urine.

---

### 29. WOUND OF THE HEART.

At a late sitting of the Royal Academy of Medicine, Paris, M. Ferrus read the particulars of a case of this kind. A lunatic, 34 years of age, pushed into his left side a long but narrow and sharp instrument, and two days afterwards was admitted into the Bicetre Hôpital, the wound being nearly cicatrized, but very painful to the touch. The pulse was small, and intermitting—the breathing laborious—and below the wound an undulating sound was heard, like the passage of blood through a varicose aneurism. The patient affirmed that he had not withdrawn the instrument by which he had inflicted the wound. General and local blood-letting was employed; but the breathing became more and more difficult every day, and the patient died on the 20th day from the date of the wound. On dissection, ten or twelve ounces of reddish sanies were found in the cavity of the pericardium, mixed with fibrinous clots. The pericardium itself was inflamed and thickened, and a steel stylet was found implanted in the parietes of the left ventricle of the heart, the point of the instrument having penetrated some lines into the cavity of the *right* ventricle, after traversing through the *left*. —*Revue Med.*

---

### 30. SYMPATHETIC EPILEPSY.

Two cases of this kind are reported in the Lancet of November 18, 1826, as occurring in St. Thomas's Hospital. The first was a boy who had been affected with epilepsy three months, having generally three or four fits *per diem*. These were preceded by an aura epileptica running from the toes of both feet up along the front of the legs and thighs, the abdomen and chest, at the top of which the two auræ met, when the epileptic seizure always took place. The progress of the aura required about a minute and produced a sensation like that arising from the crawling of an insect. Dr. Elliotson directed half a grain of the cuprum ammoniat. thrice a day, and a ligature to be applied tightly round each thigh at the commencement of the fit. The ligatures were not properly managed at first, and the fits continued; but afterwards they were more strictly attended to, and the fits ceased. The boy was discharged, but again received, the fits having returned. The ligatures were now trusted to entirely, and the paroxysms disappeared.

In the second case, the aura epileptica also arose from the feet and ascended to the neck, where the streams united, and then the paroxysm occurred. In this case, the ligatures were tried, but totally failed. It is said, however, that the height to which the streams of aura epileptica mounted, was gradually lessened. When the report closed, the fits were more severe than at first.

The aura epileptica is one of the most puzzling phenomena of this disease. The stream does not seem to run in the course of any particular nerve, artery, or muscle, but travels over all parts indiscriminately till it reaches the sensorium. The ligature, we apprehend, will never do much for the cure of epilepsy. It is too mechanical a remedy. It is very unlikely that the real source of the irritation is in the place where the aura appears to commence. The plan of stopping it by ligature has long ago been tried, and failed.

---

### 31. FISTULA IN PERINEO CURED BY OPERATION.

Mr. White, of the Westminster Hospital, has lately been successful in a bad case of the kind. The patient had been in two or three hospitals previously for a fistula in perineo, occasioned originally by a wound in that part. Mr. White introduced a staff as far as the stricture surrounding the opening, and then cut down in a line with the original direction of the urethra, till the whole of the strictured part was laid open. A gum catheter was then introduced into the bladder through the urethra, and left there. This was afterwards changed for a silver catheter, and the wound granulated over the instrument. In about a month the urine ceased to ooze from the wound, and in three weeks more the wound was closed. The strictured parts were found to be in a very indurated state when divided. The operation is not new; but we understand the case was very ably managed by Mr. White.

---

### 32. CAUSTIC IN INFLAMMATION OF THE ABSORBENTS.*

In a former number we gave a full account of Mr. Higginbottom's experience of the argentum nitratum, as an application in several surgical complaints. We have not the smallest doubt that both the internal and external use of this valuable medicine will be soon extended. The present communication of Mr. H. respects the application of caustic to inflamed absorbents. Three cases are related, of which we shall briefly notice the particulars.

*Case* 1. A woman had an ulcer formed, in four or five days, from a furuncular swelling on the forearm, near the wrist, with inflamed and irregular edges, the absorbents leading from the part, up the arm, being also inflamed. The nitrate of silver was applied to the ulcer and surrounding inflammation, as also along the inflamed absorbents. The inflammation was checked in twenty-four hours, and she was soon well. She took some blue pill and salts and senna.

*Case* 2. A girl, 11 years of age, had a fall on her elbow, 14 days previously, which caused a wound, the size of a six-pence, and from neglect, it became inflamed and highly irritable; the absorbents were also inflamed, as indicated by red lines running up to the axilla, where the glands were enlarged and tender. She had shiverings, furred tongue, head-ache, loss of appetite. Mr. H. applied the caustic along the inflamed absorbents, over the tumour in the axilla, and slightly over the wound. Gold-beater's skin was applied over the ulcer, and the other parts were left exposed to the air. No medicine whatever was given. She passed a restless night, but expressed herself relieved next day, the inflammation of the absorbents having much subsided. On the second morning, she made no complaint. The eschar remained adherent on the wound. On the third morning she was quite well.

*Case* 3. This was one of diffuse phlegmonous inflammation. A young lady presented herself with swelling, hardness, and redness of the skin, seated in the ham, and extending along the back part of the thigh and calf of the leg, which had been increasing for four or five days, without any assignable cause. The patient was feverish—the inflammation had a phlegmonous character. The lunar caustic was applied over the whole surface of the inflammation, the part being previously moistened with water. It may be necessary here to observe, that Mr. H. always applies the caustic in the solid form, and finds a slight application sufficient, if it passes over

---

* Mr. Higginbottom. Lond. Med. Journ. No. 3.

every part of the inflamed surface. On the inside of the hands and feet, where the cuticle is thick, a freer application may be made, as vesications do not so readily take place; but even in these parts, Mr. H. has not found it necessary to use it very freely. He always removes any moisture which may be left on the part by means of a little linen or lint. The young lady did not return next day, as she was desired. Three days afterwards, when sent to, she reported herself well. Mr. H. visited her, and found no vestige of inflammation or swelling. A slight vesication had been caused the day after the application of the caustic, and the cuticle was separating. No pain was experienced from the application.

The views of Mr. Higginbottom seem to derive support from the practice of Baron Larrey, as reported in our number for July Last.

---

### 33. PUNCTURED WOUND OF THE POSTERIOR TIBIAL ARTERY.

A formidable case of this kind lately occurred at Bartholomew's Hospital. A young man was wounded by a piece of iron in the calf of the leg, succeeded by considerable hæmorrhage. When received into hospital, the limb was much swollen and very tense. It was not suspected by Mr. Lloyd that the artery was wounded, and the usual means of reducing inflammation were employed. Three weeks passed away, without any reduction of the swelling, and Mr Lawrence was induced to think that a large vessel had been wounded, though he did not believe that vessel to be the posterior tibial artery. One part of the swelling softened and was opened, when a quantity of blood and pus issued, apparently from a deep source. The wound was enlarged, and the artery was felt pulsating. Mr. L. proposed amputation as the best remedy, and it was consented to. On examining the leg, a large cyst was found, whence a pound of pus and blood was evacuated. The posterior tibial artery and vein were discovered to be wounded, and this was the source of the mischief.

This case is fully detailed in No. 169 of the Lancet.

---

### 34. EXPERIMENTS ON THE BILE.—MR. MAYO.

Our readers will remember the experiments of Mr. Brodie on this subject; by which it appeared that ligature of the ductus communis choledochus of animals completely prevented the formation of chyle from the food swallowed. Magendie, in his work on physiology, states that he repeated Mr. Brodie's experiments, but with dissimilar results. This led Mr. Mayo, assisted by Mr. Cæsar Hawkins, to go over the same ground with his countryman Mr. Brodie, and his experiments, detailed in the October number of the Medical and Physical Journal, tend to confirm the conclusions to which Mr. Brodie had previously come. It will be unnecessary to do more than glance at these experiments, having already given the substance of Mr. Brodie's paper in our Journal, for June 1823, page 186, *et seq.* The experiments were performed on cats and dogs, some of which died after the operation, and others were killed; but in none of them did the least trace of chyle appear in the intestine or lacteals. Mr. Mayo thinks that Magendie may possibly have been led into error by mistaking the empty mesenteric arteries for lacteals containing chyle.

---

### 33. VICARIOUS MENSTRUATION.*

Much and unnecessary scepticism obtains, especially among the less experienced part of the profession, respecting vicarious discharges. Many will not believe in the existence of phenomena which they cannot account for—though, heaven knows, there are very few phenomena, whether of health or disease, which are capable of satisfactory explanation. The case which Dr. Barnes has related in our esteemed contemporary of the North, will doubtless be looked upon as rather fanciful, by those who do not know the multitudinous forms which hysteria and suppressed menstruation occasionally assume in females of delicate organization and susceptible nerve. We have seen many cases much more puzzling than the one related by Dr. Barnes, and some of these we have put upon record. A vicarious discharge of blood from the lungs is no uncommon occurrence, under the circumstances in question, and discharges from other organs and parts, (in impeded menstruation,) of such strange and unnatural qualities sometimes take place, as would not be credited by those who were not eye-witnesses of them. We shall now briefly state the case of Dr. Barnes' patient, which is rather prolix in the original.

*Case.* The patient is now 30. In youth, she was subject to catarrhal complaints, and occasionally to aphonia. At 14, she menstruated, but the returns were not regular, varying from three to six weeks, and the periods painful. At the age of 19, (1815) she had an attack of hæmoptysis, attended with unfavourable symptoms, as short cough, quick pulse, &c. These gave way to bleeding, blistering, digitalis, &c. Then came on symptoms of hepatitis, for which she was bled, blistered, and mercurialized. After this she remained in a bad state of health for two years, having pain in the region of the liver, dyspnœa, palpitation, indigestion, thirst, &c. The menses, in the course of this (the hepatic) complaint, entirely stopped. 1816, *aged* 21. She was again attacked with hæmorrhage from the lungs, which lasted three days. In eight months, another hæmorrhage took place, and the seizures of hæmoptysis after this came on gradually but progressively, at shorter and shorter intervals. Between 1817 and 1820, the intervals varied from six to ten weeks, and she had always the premonitory sensations of tightness across the chest, with sense of weight in the region of the heart, pain in the left hypochondrium, and difficulty of breathing, without cough. These premonitory sensations led to blood-letting, as preventive of the hæmorrhage; but no relief ever took place till the hæmoptysis occurred, excepting the sense of suffocation was removed by the venesection. Larger quantities of blood from the arm were then tried, as prophylactics, but in vain.

In 1820, soon after a pulmonic hæmorrhage, she was seized with a violent pain in the right side of the head, which forced her to spring from her bed. A discharge of clear water took place from the right ear, and this discharge is said to have returned every eight or twelve hours, for ten days. It was calculated that a teacupful was discharged at each of these times. When Dr. B. first saw her, in June 1820, the lady was much emaciated, pallid, and feeble, with pulse at 100—bowels open. We cannot follow Dr. B. in his minute details of the attacks of otorrhœa serosa, and the remedies that were tried for it. But we cannot help remarking on the somewhat confused statements which Dr. B. has made. Thus, at page 274, we are informed,

---

* Case of vicarious Menstruation, with singular Discharge from the Ear, with Remarks. By Thomas Barnes, M. D. &c. &c. *Ed. Journ. Med. Science, No.* 4.

that between the years 1817 and 1820, the intervals between the attacks of hæmoptysis varied from six to ten weeks ; and in the very next page but one, apparently under the date of 1820, Dr. B. informs us that the lady had no attacks of hæmorrhage for six years. We suppose he means, from the date of his communication at the end, but he ought to have been more perspicuous. We gather from the account, however, that after 1820, the periodical attacks of hæmoptysis were changed into sense of fulness merely of the chest, which was generally removed by opening a vein in the foot. The otorrhœa must have continued for several weeks, but when it ceased we are not informed. About 12 months ago an issue was made in her leg, from which she appeared to derive much benefit, in respect to head-aches and spasmodic pains of her extremities.

Many medical men were consulted on the case of this young lady, personally and by letter. Among others, the late Dr. Gregory was waited upon, with a written statement of this malady. The doctor said it "was a long-winded story, and a hopeless case," but in this last part the doctor was rather out of his reckoning, though we suspect he had some reason for the epithet applied to the narrative, if we may be allowed to judge by the specimen now laid before the public. In respect to the nature of the affection of the head, and the discharge from the ear, the doctor professed to know nothing—which was candid enough. He recommended attempts to restore the function of the uterus, by pediluvia and open bowels; but he had no faith in what are called emmenagogues.

Dr. Barnes adduces as a reason for considering the hæmoptysis vicarious of the catamenia, the circumstance of the former having come on "after the menses had been *suppressed*." Such, however, does not appear very clearly on the record; for we find the *first* hæmoptysis came on when the intervals varied from three to six weeks; and, moreover, that during the first six months after the commencement of the pulmonic and hepatic affection, she "menstruated three or four times." Here is a manifest inconsistency, if not a complete contradiction. It is true, the menses after this became *suppressed*, and the hæmoptysis returned; but it can hardly be justifiable to conclude that the first attack of hæmorrhage from the lungs was of a vicarious nature.

In respect to the *otorrhœa serosa*, we fear that most of those who speak candidly, will give the same answer as Dr. Gregory did—namely, that they know nothing about the matter. It is curious that Dr. Barnes never could get a sight of the patient when the aural discharge was flowing. It is possible, therefore, that some error crept in. The learned editors of the journal in which the case is detailed, have quoted examples of otorrhœa serosa alternating with the menstrual and hæmorrhoidal discharges. The same is noticed in Buchanan's Acoustic Surgery.

Dr. Barnes' case is interesting, as shewing the advantages which were *derived* from blood-letting in the foot, when the tendency to fulness in the chest occurred, and which Dr. B. considers as a much more effectual mode of relieving the upper parts of the body than bleeding from the arm. The case also shews that, in these vicarious hæmorrhages from the lungs, there is not near so much danger as in idiopathic hæmoptysis.

---

### 36. SILK-WORM-GUT LIGATURES.

In the second volume of the Edinburgh Medical Transactions, Mr. Fielding, surgeon, at Hull, has published a short paper on the above species of ligature for securing divided arteries Mr. F. remarks that Mr. Lawrence's plan of using small silk ligatures, and cutting them off very close to the knot, has not been found to answer the expectations formed of it. In a great variety of cases where he has seen this plan tried, the knots of silk were not absorbed, and were ultimately thrown off unchanged after a slow suppuration, attended with pain and irritation for several weeks or months, to the great annoyance of the patients. As the ligatures now used are attended with inconvenience, and not soluble in the fluids of the body, our author presumes that any attempt to obviate or remove these inconveniences, is deserving of attention. Time alone can prove whether the substance proposed by Mr. Fielding shall be attended with all the advantages and security which he anticipates; but he assures us that hitherto it has answered his expectations. The silk-worm-gut was suggested to our author by his assistant, Mr. Heseltein. It is much used by fishermen at the end of their lines—is apparently an animal substance—will sustain a weight of several pounds without breaking, and may be softened by being steeped for half an hour in warm water, so as to be easily formed into a knot, either single or double, as may be required. Or it may be placed in cold water over night, before it is wanted. This substance retains its tenacity long enough for union of the coats of the vessels to take place.

Twelve cases of surgical operations are briefly detailed by our author, in which this species of ligature was used with perfect success. "They prove satisfactorily, that the substance secures the vessels effectually. In no case have we seen any appearance of the knots, though carefully looked for at every dressing; and in no case has any abscess, or other inconvenience followed its use, as far as I know." It appears, therefore, to Mr. F. that the vessels are as secure—that the wounds heal sooner—and that the patients suffer less pain during the cure, than by the methods in common use. The gut may be procured at any of the shops where toys or fishing tackle are sold. We think the proposal of Mr. Fielding is worthy of further trial by operating surgeons.

---

### 37. MOXA IN PHLEGMATIA DOLENS.

In the second edition of Mr. Boyle's work on Moxa, we observe, among numerous cases where great benefit was derived from this remedial agent, a case of phlegmatia dolens, in both the lower extremities, to which moxa was applied.

The woman was a patient of Mr. Allan's; and the disease had made its appearance at the usual period after confinement. It had been treated judiciously by Mr. A. but although the symptoms were mitigated, they were not subdued. The lower extremities were enormously swollen—skin tense, hot, and transparent, with traces of extravasated blood in some places. The parts had an elastic rather than an œdematous feel, and there was much pain in the erect posture, particularly in the groins. As it was considered that a sub-acute inflammation existed, the moxa was first cautiously applied to the least inflamed limb, near the foot and ankle, and was followed by a pit or depression of the part where it was applied, together with a relaxed appearance of the surrounding skin. After this a bandage was applied, and a most profuse perspiration in both limbs succeeded. On the following day she pronounced herself relieved, and the swelling of the thigh was evidently lessened. The pain of the groin was still severe. In pursuance of Dr. Davis's Theory of Phlegmatia Dolens, Mr. Boyle applied the moxa in the line of the femoral artery in the groins, in addition to the foot and leg—and this also was attended with much advantage. The effect of applying the moxa on alternate days was tried; but it was found the disease was aggravated on the intermediate days of application. The moxa was therefore applied every day, and sometimes twice in the same day. In about three weeks the patient was able to leave town for the benefit of country air, having but little remaining swelling in her limb.

For a variety of cases in which Mr. Boyle applied the moxa with great advantage, we must refer to the second edition of his work, which is much enlarged, and is well worthy the attention of the surgical practitioner.

### 38. SUDDEN AND MYSTERIOUS DEATH.

A case lately occurred under Dr. Latham, in Bartholomew's Hospital, where a young married woman, who had been received in a febrile state, with great debility, œdema about the ankles, and pain, on pressure, in the epigastrium, was suddenly seized with something like spasm about the heart and in the bowels, a few days after she entered the hospital, and expired in the course of two or three hours.

On dissection, we understand, no organic disease or trace of inflammation could be detected in any part of the body, except near the fundus uteri, where there were some appearances of phlogosis. One of the ovaries contained hair and other extraneous matters, and was enlarged to the size of an egg.

### 39. REMARKABLY SEVERE GUN-SHOT WOUND.*

The following case is not brought forward by Dr. Boggie as an example for imitation, but rather as an uncommon instance of what Nature is sometimes capable of effecting under circumstances apparently desperate.

*Case.* Lieut. Col. L. while in the front of his regiment, in the battle of Waterloo, was deliberately aimed at by a rifleman of the enemy, and struck in the leg, by which he was felled to the ground. He was carried to Brussels in a car, and seen the next day by Dr. B. It was found that the ball had entered the fore-part of the leg about the middle, and was lodged there. It had struck upon the internal flat surface of the tibia, close to the anterior ridge, causing an oblique fracture and splintering of both bones. The colonel was 30 years of age, of slender form, temperate habits, sanguine temperament, and active mind. He was not ruffled by the accident, and the pulse was calm. An opinion was given that the leg ought to be amputated, although the operation was not indispensable at that moment. It was even proposed to make an attempt at saving the limb. There being no inflammation present, the wound was simply dressed, and the antiphlogistic regimen prescribed. Except constant pain in the fore part of the leg, there was no material change for several days. There was a splinter of bone threatening to ulcerate the skin, and causing much irritation. On making an incision over this piece of bone, a large portion of the tibia was found quite loose. This was removed, after making an incision to within an inch of the ankle-joint. The portion removed measured 4½ inches in length, involving, at one place, nearly half the calibre of the tibia. It was now the opinion that so large a portion of bone could not be regenerated, and that amputation ought to have been performed. But the patient was averse to the operation, and Dr. B. was not anxious to put it in force. The limb was simply dressed—there was no tendency to fever—suppuration came on

* Dr. J. Boggie. Ed. Med. Chir. Trans. vol. ii.

freely, and every thing went on well. The aperture by which the ball had entered was so small that it was found necessary to dilate it by the sponge tent, and several portions of fractured bone were removed. The ball lay concealed for some weeks, but was at length discovered on the fibular side, just under the skin. It was easily removed. The limb had hitherto remained in the extended posture, resting on the heel, and was now (after six weeks) changed to the bent posture, and placed on the outside. Inflammation quickly succeeded to this change, accompanied by violent fever, and threatening of gangrene. The posture was changed again to the original, and 20 ounces of blood were drawn. Cold lotions, saline cathartics, rigid antiphlogistic regimen. The inflammation and fever abated in a few days, and the patient was brought back to his pristine condition. The purulent discharge was copious but healthy. No appearance of consolidation was perceptible at the end of three months. After this period, the discharge lessened, and very soon afterwards, "osseous matter was secreted, and the union of the bones became so complete, that the leg could be moved with safety, and handled with the greatest freedom." In little more than four months the large piece of bone "was completely renewed, its regular shape preserved, and the wound cicatrized." The colonel has a very tolerable, or even perfect use of the leg, and enjoys good health. The case was seen by Dr. Thomson, Dr. Somerville, Mr. Guthrie, Mr. Bell, Dr. Hennen, and others.

The gallant officer had a narrow escape, unquestionably; but still we think Dr. Boggie was justified in the attempt to save limb as well as life, under the existing circumstances of the case. We have seen more than one instance where life and limb were preserved under circumstances at least as unfavourable as those detailed above. A case indeed lately occurred at Guy's Hospital, and is reported in No. 169 of the Lancet, where the injury was not less formidable than in Dr. Boggie's patient.

A young man had his right leg very badly fractured by the wheel of a loaded waggon going over it, and even resting some time on the limb. The tibia was broken close to the ankle joint, and it was supposed that the bone was split down into the joint. The bone was insulated from the surrounding parts for four or five inches, so that the finger could be passed nearly round it. The fibula was broken, and the soft parts were greatly lacerated. Mr. Lawrence advised amputation, but the patient would not submit to the operation. Aperients, lotions, repeated bleedings, and all the usual antiphlogistics, were sedulously employed. Suppuration and granulation went on kindly, and in two months the man was able to raise the limb, without any pain. The leg was taken out of the fracture box and laid on a pillow; but this change kindled up, as in Dr. Boggie's case, a severe fever and inflammation, so as to endanger the man's life. Happily these symptoms were got under by proper measures, and the man did well. Both of these cases shew the caution necessary in disturbing the position of the limb too soon after accidents of this kind.

---

### 40. SUBCARBONATE OF IRON.

Dr. Duparque has been testing (to use an American phrase, and by no means a bad one,) the virtues of subcarbonate of iron, in neuralgic affections, and it appears, from his testimony, that the nerves of Frenchmen are nearly as susceptible to the steeling process, so long employed in this country, as those of John Bull. Dr. D. has related a considerable number of cases in the July No. of the Bibliotheque Medicale, where the carbonate of iron succeeded, after quinine and various other remedies had failed, in facial and other neuralgiæ. We shall only give the particulars of one case, as an example.

Madame P. 68 years of age, had been afflicted, for 16 years, with suborbital neuralgia of the right side. The pains returned at irregular periods, but were seldom more than eight or ten minutes absent. The pains were of a tearing, burning character, accompanied by sneezing, lachrymation, and redness of the conjunctiva. The motions of the lower jaw, the tongue, and the pharynx, were sometimes almost annihilated, and the whole body rendered stiff, as in tetanus. All the means that could suggest themselves to some of the most eminent Parisian practitioners were tried, but all in vain, and it was contemplated to try a division of the nerve; but at this time Dr. D. administered the carbonate of iron. After four days, the intervals were lengthened to four or six hours, and the attacks were proportionally diminished.

Dr. D. remarks that this medicine has great advantages over most others; 1mo. because it is easily taken, even in large doses; 2do. because it is not capable of causing any local or general disorder in the system. None of our author's patients complained of head-ach, flushings of the face, acceleration of the pulse, or any other unpleasant effect from the medicine. It generally increased the appetite, and improved the functions of digestion.

It is gratifying to find this corroboration of the therapeutical effects of carbonate of iron in foreign countries. Indeed, we think that, next to quinine, the medicine in question is one of the most important we possess in the reduction of that morbid sensibility of the nervous system, so prevalent in this country.

---

### 41. PRINCE HOHENLOE IN PANTON SQUARE.

The prayers of this righteous prince have at length prevailed, for Margaret B——, the mother of three children, has been *miraculously* cured of an ovarian tumour, the size of a child's head, of four years standing, (and which *appeared suddenly* at the beginning) by seven bleedings from the arm in the course of nine consecutive days, in the Surgical Hospital of Panton-square. This ovarian tumour occupied "the left lower region of the abdomen"—was soft and elastic, with a distinct sense of fluctuation, and exquisitely painful on pressure, or on making a *deep inspiration.* She had symptoms of fever, and disordered bowels. After one or two bleedings, she was suddenly seized with a convulsive fit—then talked incoherently—pulse sometimes almost imperceptible—breathing stertorous—abdomen tense and tender. She was bled day after day, and took calomel occasionally. On the ninth day, when the tumour was sought for, it was gone—having been cured by seven bleedings, "without any preternatnral discharge from the rectum or vagina."

Is it in the 19th century that we are to be told such tales? The experience of mankind is the test of miracles. So said Gibbon—and so say we, especially as respects *medical* miracles. Is there a surgeon from the South Foreland to John O'Groats, who knows any thing of tumours of the ovarium, and who would, for one moment, entertain a thought that the above case was such a disease? Margaret B. had just as much an ovarian tumour, as she had an elephant's tusk in "the left lower region of the abdomen." We suppose the reporter, in this case, was one of those tender "youths of fifteen," whose education is taken under the especial protection of the RADICAL PRESS. When he grows a little older, he will distinguish those deliveries, "*à la Johanna Southcote,*" from cures of ovarian dropsy by seven bleedings.

*More Miracles.* A young man, aged 21, was admitted into the Hospital of Surgery for severe inflammation of the left eye, affecting several of the structures. Cupped to twenty ounces, and two grains of calomel, with some rhubarb and antimonial powder, to be taken every three hours. 2nd day, cupped to the same quantity, and the pills to be continued. 3d day, bled, at twice, to the amount of forty-five ounces, and ordered three grains of calomel, with one of opium. 4th day, bled *ad deliquium*. Same dose of calomel and opium. 5th day, bled again. 6th day, bled to 16 ounces. 7th day, bled and purged. The pain and inflammation now subdued.

This is a miraculous cure, and worthy of being recorded. But it is just as possible that some effusion of blood might have been spared, and the cure have been quite as speedy, had some auxiliaries been added to the LANCET, (which is the staff of life in Panton Square) in the shape of antimony, colchicum, and some other antiphlogistics. As the deep-seated textures of the eye were, apparently, implicated, some people would have given the calomel and opium in a more efficient manner, and affected the mouth in a couple of days, by which the inflammation would have been arrested. It is true, that the man is turned out of hands cured of the ophthalmia—but the lancet may, perchance, have laid the foundation of a job for the doctors bye and bye.

---

### 42. CASE OF CHRONIC DYSPNŒA.

The younger Andral, whose name is familiar to our readers, has related a curious case of this kind, which is worthy of notice.

*Case.* A young man, with glandular swellings on both sides of the neck, presented, for some time, certain symptoms of organic disease of the heart, and entered La Charité in March, 1826. His face was puffy, livid, and the eye-lids œdematous—belly dropsical—breathing short and quick, and performed entirely by the action of the ribs on each side—inability to lie down without sense of suffocation. This state of dyspnœa had continued for more than a year, and was always augmented by wet weather. The chest sounded well in every part, and auscultation could detect nothing particular in the region of the heart. A mucous wheeze was heard in various points of the thorax, with some cough, and a trifling mucous expectoration. The appetite was good, and the bowels rather relaxed, but without any abdominal pain. The pulse was natural in every respect. There were, therefore, no proofs, by auscultation, of any organic disease of the heart, although many general symptoms seemed to indicate the existence of such a lesion. There was something particular, however, in the dropsical effusion. It was confined to the abdomen, which is seldom the case in disease of the central organ of the circulation. Various therapeutical agents were employed, as local and general bleeding, blisters, diuretics, &c. During the succeeding six weeks there was no perceptible alteration. The orthopnœa was constant, and the patient was breathless whenever he attempted to get out of bed. Auscultation was repeatedly tried, but without any conclusive results. He was suddenly seized, on the 1st of May, with an augmentation of the dyspnœa, which soon became extreme, and in a few hours he died, apparently suffocated

*Dissection.* No visible alteration in the brain or spinal marrow. The heart was found perfect in every respect, as were the large vessels issuing from the heart. There were a few miliary tubercles distributed here and there in the lungs, which were otherwise sound and crepitous. The anterior mediastinum contained a mass of tuberculated lymphatic glands, through the centre of which passed the two phrenic nerves, which could not be disentangled from it, and by which they were compressed. From the exit of these nerves to their distribution on the diaphragm, they were remarkable by their grey colour, resembling that of the optic nerves in a state of great atrophy. There was no abdominal lesion of any consequence, except the serous effusion above-mentioned, and another mass of tuberculated glands in front of the vertebral column, *which pressed strongly on the cava inferior, and also the vena portæ, which they completely encircled.* On each side of the neck, from the angle of the jaw to the clavicle, there was a chain of tuberculated lymphatics, several of which were interposed between the vessels and nerves of the neck. The carotid artery and jugular vein were separated to some distance by these enlarged glands. The pneumo-gastric nerves, on both sides, were entangled in these masses, and remarkably flattened at these points. There was nothing else remarkable in the body.

*Remarks.* It is impossible not to accord with M. Andral, in placing the terrible dyspnœa and orthopnœa, in this case, to the pressure on the pneumo-gastric and phrenic nerves—in the absence of organic disease of the heart. Perhaps, Dr. Paris may be inclined to take advantage of this case for the support of some pathological opinions which he has broached in his work on diet, and which are noticed towards the close of our review of his work in this Number of our Journal. He is at perfect liberty. But we would beg him to remember that, in *his case,* there *was* aneurism of the heart, and *no* mechanical pressure on the phrenic or diaphragmatic nerves —whereas, in the *present case,* there *was* mechanical pressure on the nerves, and *no* disease of the heart. This makes some little difference. Moreover, in Dr. Paris's case, there was swelling of the limbs as well as of the body, the common consequence of disease of the heart—whereas, in Andral's case, the effusion was confined to the abdomen, and may fairly be attributed to the pressure on the cava and vena portæ.

We have marked this last circumstance in Italics, because it bears on another physiological opinion common to Dr. Paris and Dr. Yeats—namely, that the pressure of a distended duodenum on the cava, caused weakness and fluttering of the pulse. Here there was anatomical evidence of mechanical pressure on the cava, and yet it is distinctly stated by Andral, who could have no theory to support by such statement, that the pulse was always perfectly natural.—*Biblioth. Med. Juill.* 1826.

### 43. EMPYEMA.

A young man was received into Guy's Hospital, in August last, presenting a remarkable enlargement of the left side of the thorax, and a tumour the size of an orange situated between the edges of the false ribs and crista of the ilium. The man's history shewed successive attacks of thoracic inflammation, attended by the usual symptoms; and at one time he could plainly hear the squashing of a fluid in the left side of his chest. On pressing the tumour, its contents could be squeezed back. It was punctured, and 20 ounces of pus evacuated. After this, repeated evacuations took place from the tumour, and in the course of sixteen days 206 ounces were drawn off. But the cough became more harrassing—fever came on—the expectoration became purulent, and the patient died in less than a month from the operation.

The left side of the thorax was found filled with air and some remains of the fluid—lungs shrivelled up—pleura coated on all sides with a thick false membrane. An elongation of the pleural sac had taken place between the 11th and 12th ribs, forming the tumour above-mentioned. In the right lung too small vomicæ were found.—LANCET.

The size of the left side of the thorax shewed evidence of such a large quantity of fluid, that little could have been expected from its evacuation, as, in such cases, the lung of that side is generally annihilated, and it is impossible that the other lung can be dilated so as to fill up both sides of the chest—in default of which, air must get in and supply the vacuum. Still the medical officer was perfectly justifiable in giving paracentesis a trial.

We saw a man lately from a distant part of the country, who could, by a particular succussion of his chest, produce the distinct noise of water squashing about in the cavity of the pleura. There was no respiratory noise in the right side of the chest, except very high up, and that not clear. He had this complaint for several years, and was otherwise in tolerable good health. The disease seemed to be at a stand—neither increasing nor decreasing.

---

### 44. SPECTRAL ILLUSIONS; OR THE INFLUENCE OF THE DIGESTIVE ORGANS ON MR. ABERNETHY'S OPTICS.

When we heard an ejaculation of joy in the Radical Press, at delivery from an INCUBUS, we gave up all hopes of ever deriving amusement or instruction from the *facetiæ* of Mr. Abernethy again. We have been most agreeably deceived; and are happy to find YORICK once more on the tapis, "setting the table in a roar," with his lively jokes and witticisms. Whether the present series of "Tales of my Landlord," be meant to ridicule the Lecturer, amuse the public, or eke out a *subject* that is growing somewhat scarce, like *subjects* of another kind, we cannot say. But be this as it may, the lectures, as they now appear, are very curious articles, in which quaint truisms are mingled with laughable observations, and just practical rules. We shall only allude in this place to a spectral illusion of a rather extraordinary nature, with which Mr. Abernethy is troubled.

We are informed that the head of Mr. Abernethy's horse came one day in contact with his own, by which accident he had his nose broken, and, for some time afterwards, could only see "*two thirds* of an object." But even in this case, we are disposed to think the worthy lecturer's *optic* nerves were more accommodating than his *auditory*—for, to this day, the *latter* will not listen to more than *one third* of a story—unless, indeed, the story be his own. Thus, in looking up at his own name in a shop-window,

A—BER—NE—THY, he could only see as far as the NE (knee) for the THY (thigh) was quite out of the field of vision, *(a laugh.)* What witty dogs are these nightmares, or rather their exhibitors!

Mr. A. has occasionally, some curious "errors of action, or inactivity in parts of the retina," resulting, we should imagine, from his "digestive organs." He can, when looking in a glass, see the upper part of his head, but "he never saw (says he,) that he had any mouth or jaw"—all being blank between the nose and shoulders.* We are most happy to think that these are only spectral illusions, and hope the worthy lecturer will long continue to convince the world, that he has a mouth of his own to tell his pupils so many droll stories, and a MOUTH-PIECE in some reporter to retail them to the public at large.

* We understand that a still more curious *illusion* occurs when Mr. Abernethy looks at a patient—for then he can see nothing but the *tongue*—all the rest of the patient's body being a blank.—*Rev.*

45. ERGOT OF RYE IN UTERINE HÆMORRHAGE.

M. Goupil, after relating a case where the secale cornutum hastened delivery, proceeds to the statement of another case where it apparently tended to arrest a uterine hæmorrhage.

*Case.* Madame F. the mother of three children, was delivered of a fourth, after half an hour's expulsive pains. The placenta was removed by a midwife, and, according to her own account, without any force. A profuse hæmorrhage succeeded, and M. Goupil was summoned. Cold applications, and the introduction of the hand into the uterus failed to arrest the flow of blood; and, in this predicament, our author administered twelve grains of the ergot. In ten minutes there was a powerful and painful contraction of the uterus. The hæmorrhage was arrested, and did not return; but a second dose was administered, by way of precaution.

We much wonder, after the evidence which has been afforded, of late years, respecting the power of this substance over contraction of the uterine fibres, that it has not been frequently tried in uterine hæmorrhage.—*Biblioth. Juillet.*

46. VESICAL FISTULA.

In No. 167 of the Lancet, a case of this kind is related, on which we have a remark to make. The patient was originally under Mr. Burrows, an intelligent surgeon in Holborn, for a swelling just below the umbilicus, which terminated in suppuration, and was opened by Mr. Stanley. Ill-conditioned pus was first discharged, and soon afterwards urine came through the opening. This discharge had continued about two years, when he was received into Guy's Hospital. "When the patient strains, or calls the abdominal muscles into action, a fluid is discharged from the opening, which is undoubtedly urine. A small quantity of urine only is discharged in the natural way." Sir Astley Cooper examined the case in Guy's, and concluded very justly that there was a fistulous communication between the abdominal aperture and the bladder. But the reporter puts an expression into Sir Astley's mouth which we are convinced that distinguished surgeon never uttered, namely that—"it was almost inexplicable that the urine which was discharged into the bladder, at a considerable distance below the fistulous opening, should, as it were, ascend against its own gravity, and be expelled from the aperture."

If the reporter were well acquainted with the laws of physics or physiology, he would be aware that there never is any cavity in the bladder, the parietes of that organ being alwayst n contact with their contents (allowing for the rugæ) whether there be a spoonful or a quart of urine in the bladder. The fistulous aperture, therefore, supposing it to be in the very fundus of the bladder, is always in communication with the urine; and, as there is no sphincter to be overcome there, when the abdominal muscles are thrown into action, where is the wonder that the urine should issue from the external aperture?—*qua data porta ruit.* The patient is in the same condition as a man whose bladder is punctured above the pubes, and a tube left in the wound. In such case, the water is always dribbling through the canula as it is secreted, unless the tube be kept corked. We knew a gentleman who passed all his urine in this manner for two years or more, and, after all, the urine came through its natural channel ultimately, and the artificial opening above the pubes healed. The man in Guy's Hospital should have a catheter kept in the bladder, and pressure made on the fistulous opening in the abdomen. This would give him a very probable chance of cure.—*Chirurgus.*

### 47. EXCISION OF PART OF THE MAXILLA INFERIOR.

Mr. Lizars, of Edinburgh, has lately added another wreath of laurel to his brows, by the operation above-mentioned. The patient was a robust man, 38 years of age, who had been operated on, two years previously, for cancer of the upper lip. Several months afterwards, a lymphatic gland at the base of the inferior maxilla enlarged and became adherent to the bone. This had lately become rounder, conical, the size of an orange, so as to completely lock the two jaws. In short, the disease was pronounced to be osteo—sarcoma, and considered by Mr. L. as incurable, except by the entire removal of the diseased bone. The operation was performed on the 16th August, in the presence of Dr. Scott, of Cupar, Dr. Duncan, Dr. Poole, Dr. Borthwick, &c. The steps of the operation are minutely detailed in the October Number of the Edinburgh Journal, and do great credit to the operator. In the early stage of the operation, a multiplicity of arteries sprang, and required the ligature. Mr. L. then proceeded to remove the bone, first rendering it rudely clean at the symphysis, where the disease had extended so far as to involve the four incisor teeth. He attempted to snap the bone with Mr. Liston's nippers, but was foiled, and obliged to saw it through. He next detached it by sweeping with the scalpel, its cutting edge kept towards the chin and trachea. The lingual artery with some others sprang at this time, but the bone being removed, they were easily secured. Mr. L. carefully examined the surface of this extensive wound, removing every apparent vestige of disease. The wound was then sponged, the lips approximated, and proper dressings applied. The operation was rendered exceedingly complicated and difficult by first attempting to remove the tumour and preserve the bone. This was found impracticable, because a part of the tumour was formed by the bone itself, and incapable of being detached from it.

The symptoms which succeeded this formidable operation were not more severe than might reasonably have been expected, and the recovery may be said to be complete. The patient can swallow any pulpy mass—the gap is exceedingly small, about the twelfth of an inch in breadth, and one inch in length. He feels no pain in any part of the wound, and both the cut ends of the bone are covered with granulations. He sits out of bed, and could walk about the room, at the date of report.

The operation is exceedingly creditable to the intrepidity and dexterity of this accomplished surgeon.

In a late sitting of the Parisian Academy, M. Dupuytren presented three patients on whom he had performed the operation in question. This talented surgeon, when on a visit to the Hospital of Invalids 14 years ago, observed that some of the soldiers had had portions of the lower jaw carried away by gun-shot wounds, and yet survived the accident. He, therefore, determined to perform this operation in cases where tumours were adherent to the bone, and incapable of being removed otherwise. In the case of one of the patients now presented, he divided the jaw bone at the symphysis, and amputated a large portion of the bone, including several molares. The soft parts were brought together, the ends of the bone approximated gradually, and, in twenty-five days, the man (who was a hackney-coach master) was able to resume his occupations. M. Dupuytren has now performed this operation seventeen times, fifteen of which proved successful.

---

### 48. ORTHOPEDIA.

A female had her leg fractured, and being badly attended, in a chirurgical point of view, the limb, at the end of four months, was so crooked that she was obliged to walk on the exterior edge of the foot. M. Desgranges, of Lyons, undertook to remedy this defect, and by means of mechanical extension of the member, and well applied pressure on the protuberant angle of the fracture, the most perfect rectitude of the leg was obtained.—*Transactions of the Medical Society of Lyons.*

### 49. RETROVERSIO UTERI.

An interesting case of this kind is related in the Ed. Journal of Medical Science, No. 4, by Mr. Wise, the author of an able paper on the trephine, in a former number of this Journal. The woman was 35 years of age, well formed, and of healthy constitution, at the full period of her fourth pregnancy. When Mr. Wise attempted to introduce his finger, he found the canal completely blocked up. With some difficulty he got his finger upwards and forwards to the top of the pubic arch, and there felt the os uteri dilated, and a foot presenting. On examination per rectum, the head of the child was distinguished low down in the rectum, filling the hollow of the sacrum. A consultation was requested, and, in the mean time Mr. Wise learnt that, between the third and fourth month, the flow of urine became obstructed, for which the woman took a variety of nostrums, without relief. In this state, a surgeon residing nine miles distant was employed, and drew off her water once a week, there being a constant stillicidium and great distress. By pressing on the head of the child through the rectum, and laying hold of the feet and dragging it in that direction, the delivery was effected, though with considerable difficulty. After this, the patient expressed herself as greatly relieved; but fever came on, and she sunk on the 15th day.

It is difficult, we imagine, to say whether the obstruction of the urine, at the time above-mentioned, was the cause or the effect of the retroversion. A full bladder may cause retroversion—and retroversion may cause such pressure on the neck of the bladder by the tilting forward of the os uteri, as to obstruct the passage of the water.

For numerous interesting remarks on this subject, by Dr. Campbell, of Edinburgh, through whom the communication was made, we must refer to the original paper in our contemporary.

### 50. PHAGEDENA GANGRENOSA.

A wretched female from Swan-alley, near East Smithfield, was received into Bartholomew's, with sloughing phagedena of the right labium, which had commenced, five weeks previously, as a pimple, which vesicated, and gradually enlarged to its present size, three inches by one. The surface was irregular, and covered with a fetid, pulpy, thick slough, with dark brown discharge, and a halo of surrounding inflammation. The edges were abrupt, and the surface of the sore was dotted with angry-looking points, considered by Blackadder as characteristic of the disease. The peculiarly fetid odour which usually accompanies this sore was here very annoying; but it was not attended with that darting, though intermitting pain, considered by Mr. Welbank as inseparable from the disease. The right labium was destroyed. The general health did not seem much affected—pulse natural—tongue clean —no look of distress. Mr. Lawrence, *therefore,* considered all constitutional treatment as unnecessary, and only directed the application of concentrated nitric acid, the sore to be afterwards covered with dry lint. She did not complain much of this application. An opiate. In the course of two applications, some parts of the ulcer began to wear a more healthy appearance, but, generally speaking, the disease was advancing. Three days more trial was given to the acid, but without much effect. It was, therefore, given up, and opium, internally and externally, was resorted to. This plan was not effectual, and the acid was again used, in conjunction with the opium, when success finally crowned the surgeon's efforts.

This case is well reported in the LANCET.

But we have a remark to make on it. We question the principle on which Mr. Lawrence determined not to have recourse to constitutional treatment, *because* there were no indications of constitutional indisposition. Now, every man of observation must have seen numerous instances where there was no other obvious disorder of the system than what might be suspected from the bad character of a local sore. Such was the case, we think, here, and, in fact, Mr. L. was obliged to lull constitutional irritation before he could master the sloughing ulcer. The pulse, the tongue, and the countenance may not indicate disorder of the system, and yet many of the secretions and excretions may be unhealthy. The surgeon, therefore, should not be too confident in his caustic, or even his scalpel, but wisely combine general with topical remedies.

---

### 51. ON MERCURY.*

The writer, from experience of nearly twenty years, offers a few observations at variance with those of a work on Indian Diseases. That author, professing to take for his guide the appearance of the evacuations in dysentery, (a useful guide certainly) asserts, that to continue the use of mercury for months, "*or even till ptyalism be produced, is most injurious to the constitution.*" He also thinks mercury " more beneficial in chronic than acute dysentery;" and that " he is contending against the bulk of opinion in India."

The author is contending against the positive result of practical experience; for the *records of practice* will prove, that the cessation of fever, in cases where mercury has been used for its removal, has taken place at the moment at which the register states the *salutary* effect to be visible on the gums—thus, " gums sore, the patient had not his usual attack of fever." In acute dysentery, the records will shew, that with the ptyalism, that is, immediately on its being established, " improved secretions" forms an article in the entry, and in acute hepatitis, after the most active depletion, and other auxiliaries. No one would pronounce his patient safe, till he had evidence of the *salutary* effect of the mercury on the gums. How, then, is it pernicious or injurious to secure ptyalism? That perseverance " for months," even under the *salutary* effect, is injurious, and disgraceful to the practitioner who enforces it, is undoubtedly true; but there is an effect of mercury which the writer has seen mistaken for the salutary effect, and the consequence of which has been death. In some from ignorance, in others from inattention, the production of *a sore mouth*, and continuing the soreness, is all that is sought. It is well known, that, when mercury does *not* produce its *salutary* effect, it does produce ulceration and a clammy, viscid state of secretion, totally unlike true ptyalism; with this there is great general irritability; yet the writer has seen a patient die *from perseverance under such circumstances;* undoubtedly from irritation, as, on examination, there was no sufficient cause of death, *except the treatment.* Even where, from abscess having al-

* On the Effects of Mercury in Diseases of Warm Climates. By MEDICUS.

ready formed, the constitution will not take on the salutary influence of mercury, the earliest opportunity should be taken to discontinue it; as well as in the case of *natural insusceptibility of the salutary effect.*

Of the positive, and, it may be called, *periodical* good effect of the action of mercury in acute dysentery, the writer has already asserted his conviction, and thinks there can not be found, in medical reports, any such evidence of its effects in *chronic* disease.

The object of these observations is, to urge the junior practitioner to secure that state of gums, *which is almost* invariably the index of the establishment of that mercurial action on which success depends;* (in this the previous active depletion will greatly assist) and *to guard him very seriously against perseverance in the use of mercury, where the symptoms shew the constitution to be adverse to its favourable or salutary effect.* In such cases, the writer believes, immediate change of climate, with the use of nitrous acid, the most advisable mode of treatment.

A combination of antimony with mercury will succeed sometimes when mercury alone will not.

*Nov.* 1826. MEDICUS.

---

### 52. CHRONIC GASTRITIS.

On this important subject we have brought forward much interesting information lately, from continental practitioners. The subject of the present short notice, is a case related by Mr. Brown, in the second volume of the Edinburgh Transactions.

A female aged 40, came under Mr. Brown's care in August 1825. She had been exposed to cold about a month previously, while in a state of perspiration, and had been ailing from that period. She had been affected with diarrhœa, for which she had, as is the usual routine, taken *purgative* medicines. Mr. B. prescribed something which is not stated, but which was attended with benefit. On the 7th September the following symptoms were noted :—pain in the stomach, *immediately after taking food,* without vomiting, "or at least without constant vomiting;" and pain on pressing the epigastrium. These symptoms continued unabated till the patient's death, which took place on the 23d November of the same year. The leading and unvarying feature was pain, sometimes of a burning kind, on receiving food or drink. Vomiting was sometimes solicited by the patient to relieve the stomach. The bowels were generally costive, with occasional diarrhœa—pulse seldom above 90—perspirations troublesome, but no other symptom of hectic fever. The appetite was good to the last, but she ate very little. "*She said she was sure she would be quite well if she could do without food.*" The emaciation was extreme. A few days before her death, a small circumscribed swelling was observed, lying over the region of the pancreas, having the regular pulsation of the aorta. She had been twice or thrice leeched and blistered, and had the antimonial ointment applied, without any marked good effect. She twice took the blue pill to the extent of slightly affecting the mouth, and said she was easier both times. Bitters and chalybeates, as might be expected, produced no beneficial influence. "Opium gave relief in all stages of the complaint."

*Dissection.* The mucous membrane of the stomach was every where thickened and vascular. There was no disease in the liver, intestines, spleen, pancreas, or any other organ.

---

* The writer has seen two instances of *small* abscess forming in the liver, where the patients were under the satisfactory effect of mercury.

The author very candidly acknowledges that the treatment, in this case, was too inert, especially in the beginning. There can be no question that this was a case of simple dyspepsia, or morbid irritability of the mucous membrane, at the commencement, passing ultimately into chronic inflammation. Had this patient been placed but on the mildest farinaceous food, reduced to the quantity that could be taken without pain or other uneasy sensation, and every thing stronger than water prohibited, she would have got well, without any medicine whatever. Even when chronic inflammation was established, as it was most unequivocally, the same plan of diet would have been the main item of cure. Leeches and counter-irritation should then be *weekly* employed, and mild anodynes, especially hyoscyamus with minute doses of blue-pill, to keep the secretions in order, should be given at intervals of not more than six or eight hours.

Many are the cases in which *irritation* passes into *inflammation*, from the want of attention to diet, and from the injudicious use of tonics and stimulants! But the practitioner, it must be owned, has not often such influence over the patient as to enforce the proper system of diet. The case related conveys a very instructive lesson, and it would be well if patients could be made sensible of the danger they run in the indulgence of their appetites.

### 53. COMPOUND DISLOCATION OF THE ANCLE-JOINT.

A very bad case of this kind lately occurred in the hospital practice of Mr. Tyrrel, and was treated successfully. A man of middle age had his foot caught under the gate of the swing-bridge in the London Docks, by which the ancle-joint was dislocated, and the integuments lacerated so as to expose the joint. The tibia protruded two inches or more, and was fractured across, the extremity being separated from the rest of the bone. The other structures about the articulation were much injured. The fibula was also fractured at some distance from its lower extremity.

Mr. T. removed the separated portion of tibia, and reduced the dislocation, bringing the soft parts together, as well as he could, and retaining them in co-aptation by proper dressings. The limb was then laid, with the knee bent, in a hollow splint, another short splint being placed along the inside of the leg. Evaporating lotions were applied. The febrile excitement was not great; and, although some sloughing took place, suppuration was established, granulation ensued, and the man did well.—Lancet.

### 54. SLICING STRUCTURE IN QUEST OF FUNCTION.

We are not among those who decry experiments on living animals; but we think the attempts which have lately been made, and so often repeated, of slicing and cutting the brains of animals with the hope of detecting the functions of its different parts, are hardly justifiable by the prospect of any advantage that may thence result. Dr. Hertwig, of Berlin, has carried on these experiments, of late, very freely; and the only thing curious that we can see in them is, that, if experiments on hens, pigeons, and rabbits, can determine the localities of the mental faculties, or the seat of the soul in the brain, the phrenologists are all in the wrong. He concludes, that the hemispheres of the cerebrum are the "*true seat or the central point* of all the perceptions, of consciousness, of understanding, and the will"—that these powers of the mind and of the senses have a *common seat* in the hemispheres—and yet that "the consensus communis is not dependent on the hemispheres alone, *since it remains after they have been destroyed*." If this be not converting the obscurum into *obscurius*, we know not what is!

Dr. Hertwig will be a stumbling-block to the materialists—for if the faculties remain after the brain is destroyed, we need no farther argument for the immateriality of the soul!

---

### 55. MR. ABERNETHY ON SPINAL DISEASES.

Mr. A's lecture on this subject, as reported in one of the late numbers of the Lancet, contains some opinions and practices which deserve a few comments.

Mr. A. dislikes the "*genteel*" term *concussion*, as applied to the spine, because it merely conceals our ignorance; and he, therefore, gives it the more scientific appellation of "*jarring*," which, in our humble opinion, adds nothing at all to the elucidation of the accident. Mr. A. believes that dislocation, without fracture, can only take place in the cervical portion of the spine. He says we cannot reduce these fractures, for if we pull at the spinal column, for the purpose of extension, we may "*jerk the medulla spinalis asunder.*" He, therefore, advises his pupils to keep the parts still. Most of these cases are dangerous; but our lecturer gives us the pleasing consolation that he has seen "many cases of recovery, even where the fractures were in the neck." Mr. A. has certainly been much more fortunate, in this respect, than his neighbours. He relates the case of a medical student, who fell into a gravel-pit, and fractured the spine, about the middle of the dorsal portion. In two months, he was so well as to be able to sit up and play cards, &c. but all below the fracture was completely dead. Mr. A. does not condemn the operation of trephining the spine, as practised by Mr. Tyrrel, but thinks it should not be attempted till all symptoms of inflammation have subsided—the same as in cranial fractures, with depression and compression. Now, if we wait till the symptoms of inflammation have subsided, it will be too late to save the spinal marrow from compression; and, therefore, if the operation is to be attempted at all, let it be done before the inflammation begins. It appears that Mr. A. did actually propose such an operation himself.

On distortions of the spine, Mr. Abernethy is, as usual, very droll. He says these "are not diseases but deformities;" yet in the same column he informs us that "he does not see these curvatures happening, *except where there is some constitutional disorder*." If this be the case, there surely must be some connexion between the constitutional disorder and the spinal curvature. Particular postures, then, cannot be the sole cause of these deformities, since such postures are assumed occasionally by all young people. The fact appears to be, that the spinal column and its muscles partake of the constitutional disorder, and give way under postures or habits which, in perfect health, would produce no distortion. The whole therapeutics of the Lecturer may be comprised in a single line—*alternate rest in the horizontal posture, and active exercise.* Where there is anterior or posterior curvature, dependent on diseased vertebræ, rest without exercise must, of course, be enjoined. In these precepts the novelty is not of the very first order; yet they are made to cover a vast quantity of paper in the pages of the Lancet.

---

### 56. TREPHINE, IN INJURIES OF THE HEAD.

We have observed a paper in the New York Medical and Physical Journal for March 1820, which, as it bears upon the subject we discussed in Art. 5, of the Periscope of this number, we shall notice here. It is a case of epilepsy, after fracture with depression, cured by trephining.

*Case.* Mr. D. æt. 46, received, in the year 1811, a blow from a bar of iron, which wounded the scalp and fractured the frontal bone. He was taken to the hospital, but there were no *symptoms*, and he was not trephined. In a few weeks he left the hospital quite cured, but epileptic fits, after a short time, came on. At first there were considerable intervals between these accessions, but latterly he had two or three in the day. When Mr. Rogers, the narrator of the case, saw the man in 1825, he was almost idiotic—had a voracious appetite—and complained of pain over the left superciliary ridge of the frontal bone, where there was discovered a large cicatrix and " a depression sufficiently large to admit the extreme point of the finger." The pain extended down the neck and left arm ; the eye of that side was diminished in size, and its vision much impaired ; the memory was almost destroyed. Under these circumstances, Mr. Rogers determined on removing the depressed bone, and on the 7th July, 1825, with the assistance of Professor Mott and Dr. Ring, he proceeded to apply the trephine. The operation however was difficult, in consequence of the saw having to pass through the upper part of the frontal sinus, and, unfortunately, the dura mater was cut through for one half of the circumference of the circle. A portion of the internal table was found to have been fractured, separated from its attachments to the frontal plate, and driven back upon the substance of the brain. All went on well, and there was no return of the fits until the 25th day, when, in the absence of the nurse, the patient overloaded his stomach with food. A fit, almost like apoplexy, was the consequence, and this was followed by a severe inflammation of the brain. This was checked by active depletion, and on the 20th August, he was discharged cured. Nine months afterwards he had had no return of the fits, and his memory had almost recovered its strength.

Mr. Rogers has appended to this case some observations which prove him a stanch anti-trephinist. He says, that, in simple fractures with depression, antiphlogistic treatment alone will be found for the most part sufficient, and in this, perhaps, he is right. But he next attempts to subvert the distinction, which Sir A. Cooper has so ably elucidated, between simple and compound fractures of the cranium; and in this we are sure he is wrong. The mode in which he controverts this position is singular enough. He says—" the only difference in the two cases proceeds from the bone—in one case being exposed to the air, while in the other it is covered by the integuments." This is the *only* difference to be sure, but it makes a vast deal of difference for all that. If all the " symptoms" arose from mere compression, as we have again and again observed, then, perhaps, this *only* difference would be but a trifling one ; but since they arise, for the most part, from irritation, and since *we know* that the access of air to parts naturally excluded from it, is a great source of irritation and inflammation, so we can easily account for this *only* difference proving a most material one.

The next argument Mr. Rogers brings against the trephine is the stale one of cases doing well without it. No doubt many patients, with severe affections, will do well without the *use* of the doctor at all ; but the doctor is a very *useful* personage notwithstanding. In fact we think this a most childish argument, for it is not on exceptions that we are to ground the rule.

Mr. Rogers adduces two cases of compound fracture of the cranium with depression, in which active depletion alone conducted to a complete recovery.

In the first, the fracture was of the right side of the frontal bone, and the depression four inches in circumference. The patient lost by the lancet 161 ounces of blood in the course of six days.

A case of extensive fracture of the cranium occurred lately at St. Thomas's Hospital, and is recorded in the number of the Lancet for October, 21st. A boy, æt. 13, was brought into Hospital, Sept. 25th, having received a blow on the head from a falling pole two hours previously. There were some of the symptoms of concussion present, drowsiness, &c. but he could answer questions correctly. At and about the posterior and superior angles of the parietal bone, was a circular fracture three inches in diameter. The bones were divided into several portions, one of which was depressed full half an inch. The scalp above was puffy, but not wounded. Over the right frontal sinus the integument was torn in consequence of the lad's having fallen on that part. He had frequent retching, and vomited occasionally matters streaked with blood. The symptoms of compression not being present, Mr. Green, under whose care the boy was, determined not to operate. Five grains of calomel were taken immediately, and cold lotions applied to the head. V. S. ad ℥xij. *26th.* The scalp had become much swollen. Pulse 40 and oppressed. Severe pain in the head—incoherence—V. S. ad ℥xvj. Five grains of calomel immediately—house-medicine—an enema. During the bleeding the pulse and the boy felt himself relieved. For the next two days, he continued much in the same state—there was occasional incoherence—but he could generally answer questions—the pulse was irregular, ranging from 48 to 60 and upwards. He was bled twice, and on the 29th was evidently improving—the pain in the head had disappeared—the skin was cool—the bowels had been freely opened, and the heaviness of the countenance was gone. On the 13th October, the patient was playing about the ward.

The reporter of this case seems to have thought it a mighty strange thing that there were no "symptoms" immediately after the receipt of the injury, but, perhaps, when he has seen a few more cases of fracture of the cranium, he will not consider the circumstance so very extraordinary. Now contrast this case with the one we have related in Art. V. of the Periscope, as occurring at another hospital, and we think that any unprejudiced person will allow the distinction of fractures of the skull into simple and compound correct. In this case the scalp was not wounded, and although the depression was certainly more extensive than in the other, the symptoms of re-active inflammation were by no means so violent. It will be said indeed, that in the former case the brain itself was injured, but experience tells us that if the foreign body, the bone, be removed, the mere wound of the brain is by no means necessarily fatal. The subject is one of such paramount importance that we shall, as opportunity offers, return to it again.

---

### 57. SPASM OF CANALS.*

Since Dr. Monro's paper on this subject, published in the 3d No. of our junior Northern contemporary, and reviewed in our 10th Number, Mr. Allan has communicated to the author, two facts illustrative of the spasmodic power of the urinary bladder. The first was in a case of lithotomy, operated on about ten years ago, where one stone being extracted, and another found to exist in the bladder, the operator repeatedly introduced the forceps, without being able to effect his purpose. This arose, Mr. Allan avers, from the spasmodic contraction of the bladder, for, on introducing his finger, he found the stone firmly held in its situation above the pubes, by the vesical spasm. The extraction was deferred till the evening of the third day,

---

* Dr. Monro. Additional Observations, &c. Ed. Journ. Med. Science, No. 4.

when it was easily hooked out by the finger alone. The patient recovered without one bad symptom.

The next case was that of an irritable boy, three and a half years old, who was operated on, June 6th, 1826. Mr. Allan anticipated a spasmodic affection of the bladder, and, therefore, prepared the little patient by previous anodynes and aperients. A dose of laudanum was also given on the morning of the operation. Still the dreaded spasm took place. On introducing his finger, Mr. A. readily felt the stone, but whenever the forceps were introduced, "it *disappeared*"—or rather, we should say, *vanished*. This repeatedly occurred, and Mr. A. gave up the idea of extraction by metallic instruments, and managed by gentle means to hook out the stone by means of his finger and a small lever constructed for the purpose.

We have no doubt but that this spasmodic contraction of the bladder is sometimes the cause of embarrassment in extracting calculi. From the facts before us it does not appear that precautionary measures, in the way of lessening irritability of the part, are to be much depended on. There is one comfort in Dr. Monro's doctrine—namely, that all difficulties in getting a stone out of the bladder, in future, can be conveniently laid to the charge of spasm. We all know that, in every house, there is a certain personage, better known to the servants than to their masters, whose name is "NOBODY," but who is the reputed author of every breakage, spillage, and mischief committed in the house, from January till December. This personage too, like the spasm of the bladder, is never seen, though very plainly *felt*, not only at the time he plays his pranks, but afterwards when the mischief is to be repaired. *Sat verbum sapienti.*

### 58. MR. LAWRENCE'S LECTURE ON RE-MODDELLING THE MEDICAL CONSTITUTION.

This gentleman has discovered that no accurate line of distinction can be drawn between physic and surgery—and in this we agree with him. We also concur in his opinion that the education of the physician and surgeon should be the same in kind, though perhaps differing in degree—that is, that each should study, as far as his circumstances will allow, all the branches of medical science. On this point, we think it is impossible for men of observation or reflection to differ, unless they labour under a bias of their judgment. But when Mr. Lawrence contends that there should be no division of the healing art, whether as respects tuition, practice, or rank —we differ from him *toto cœlo.* Instead of the recent progress of medical science giving "a fatal blow" to the divisions of labour, either in teaching or practising, we see these divisions almost annually increasing; and we are really astonished how Mr. Lawrence can reconcile what he says in the lecture-room, with what he sees every day around him. What would be thought of the state of brain in that man who should open a lecture-room in this metropolis, for the purpose of teaching anatomy, physiology, pathology, therapeutics, surgery, midwifery, botany, chemistry, materia medica, medical jurisprudence, &c.? He would soon be put under restraint in the New Bedlam. Did it never happen to strike Mr. Lawrence's mind that, if a gentleman, after studying all, should cultivate *one* of the above branches with more care than the rest, he might be able to teach that branch with more success? And if he looked about him, might he not perceive that the same observation applies to practice. Would any man (any general practitioner, for example) who had a child seized with typhus fever, dream of sending for Sir Astley Cooper to give his advice—or run past Sir Astley's door, in Spring Gardens, to summon Dr. Maton, if another child had his leg jammed off by the wheel of a carriage? Or, if craniotomy was to be performed, would he dispatch a messenger for Mr. Lawrence, notwithstanding that gentleman's very profound knowledge of the brain? No, verily;—and why? Because, although the student may take lessons from all the different teachers of the different branches of medical science, it is his experience afterwards which alone gives him *knowledge*, the former being *learning*, which is a very different thing. Now the man who lays himself out for one department, as for example, operative surgery, will have greater experience than others in that department—he will be more expert from experience—and he will *always* (till the end of the chapter) be sought after by the public, on account of his expertness. The same may be said of all other departments.

Medical science is too extensive for *all* its branches to be either studied or practised by one individual, with that minuteness and success which commonly attend the study and practice of a single branch. Every year this truth is becoming more and more conspicuous. Look to Mr. Bennett's lecture. There we find that *one* of the branches of medical science (anatomy) requires "a number of individuals, *each devoting himself to the cultivation of particular parts of it*," before perfection can be attained. For all practical purposes, then, there is sufficient distinction between physic, surgery, and midwifery, for the metropolis and every large city to afford practitioners devoted to one or other of these branches. Still, for general use, there must be general practitioners; but he who can study and practise *every branch* of the profession, and arrive at as much perfection in *all* these branches, as he who *only* studies *one* in particular, must have a genius equal to that of Mr. Lawrence himself, which, it will be, of course, acknowledged, is impossible.

Mr. L. expends a monstrous deal of wit in his dissection of the College of Physicians, "instituted by a monarch more famous for lust than medical knowledge"—of that young whelp, the College of Surgeons, "fortunately for *us* entirely destitute of power"—and of that College of Apothecaries, "who keep a shop for the sale of drugs." Like a true radical, he is dissatisfied with all existing institutions of this kind, and proposes the appointment of "an examining *body*," (in which *somebody* would doubtless take a leading part) empowered to test all candidates for medical practice, "without any regard to the present artificial distinctions;" which examination "should authorise the individual to practise any or all parts of medicine, *and none should be allowed to practise without it*." Thus, then, according to this Utopian plan, the metropolitan physician and surgeon, and the village apothecary and accoucheur, must undergo the same examination, and consequently must have the same general and medical education! This is equality, with a vengeance! Now, if Mr. Lawrence had given himself the trouble to think for one small minute before he spoke, he would have perceived that such an equalization would place the necessary acquirements above the reach of a great proportion of general practitioners in the country—and that it would reduce the said acquirements to too low a standard for those who aspired to honours and distinctions in cities and in the metropolis. The consequence of such an attempt at equalization would be, a degradation of the upper classes of medical society—an exclusion of the lower classes—while it left the middling classes just where it found them. If the examination were at all impartial, and adapted to the wants of society, it must be calculated at a *medium* rate—and, consequently, it would be too low for the one extremity, and too high for the other. But the fact is, that Mr. Lawrence's scheme in medicine, is just what the scheme of Hunt and Cobbett is in society at large—equality. The one scheme is as feasible as the other, and both will be accomplished about the same period of time.

### 59. PUZZLING POINTS FOR A COURT OF CONSCIENCE.

It is well known, that all members of the Royal College of Physicians, fellows and licentiates, take an oath not to derogate from the honour of the College by any word or act on their parts. In the recent paper-war respecting the Hunterian Museum, a fellow of the College has charged a licentiate with violation of his oath, in daring to *censure a censor*, while the latter was in the execution of his *public duty*. The licentiate repels the charge, averring that, as a public journalist, it was his duty to criticise the public conduct of men in office; and he moreover charges the fellow of the College with a much greater derogation from the honour of the College, by not only publishing letters in the Radical Press, but publicly acknowledging himself to be an *anonymous writer* in that vehicle!

We understand the Bench of bishops are to decide this knotty point. In the mean time, we may be permitted to observe, that we have not met with an instance of such daring intrepidity, on record, since the descent of Orpheus in search of Eurydice, as has been evinced by a fellow of the Royal College of Physicians, venturing his name in the pages of the Radical Press. We think we may assert, that the gentleman in question, who has otherwise deserved well of his brethren, will never again degrade himself, by communication with a Press which openly avows hostility to every established order of things in the profession.

---

### 60. EXHUMATION—FUMIGATION.

In consequence of the great scarcity of *subjects*, and the important discovery of Labarraque, respecting chloruret of lime, as a disinfecting agent, a joint-stock-company has been formed, for the exhumation of all those dead, the term of whose patent coffins has expired. The experiment has lately been tried, with great success, by the new company, on the body of the late Mr. J. C. Saunders. The exhumation and *post mortem* investigation lasted upwards of six weeks; and was conducted with the greatest *sang froid* by the company's resurrection and dissection men. We understand, however, that, notwithstanding the power of the chloruret, the offence against the sanctuary of the grave appeared to the bystanders to be "rank, and smelled to Heaven." The company are sanguine in the hope that TIME and HABIT will reconcile the olfactories of its customers to these "unsavoury suits."

---

### 61. NEW REGULATIONS OF THE COLLEGE OF SURGEONS.

In our last Number we published these regulations, but had not time for any observations on them. This defect has been amply supplied by the Radical Press, which seems to have exhausted every term of vituperation on the new regulations. We do not wonder, however, that the Press should be in a passion, when such men as Cooper, Abernethy, Cline, Home, Guthrie, &c. are conspiring to rob the Radical Press of a portion of its daily bread.

"We will not say that they are rogues by nature, but do not hesitate to assert, that their proceedings are characterized by a most roguish aspect. A thief at the Old Bailey would act in a manner precisely similar."

To this mild and courteous remonstrance are added some choice epithets, as "corporate tyrants"—"gross injustice"—"deeds of infamy"—"acts of cowardice and meanness"—"floundering imbecility"—"slippery reptiles"—"maggots of hospital corruption," &c. &c. &c.

After this exquisite specimen, which shews the Augustan age of medical literature in which we live, we shall make a few calm observations.

We are not "toad-eaters," or "sycophants" of the College. The regulations that have been rescinded never had our approbation,—quite the contrary; but we cannot put ourselves in a fury because we see the *liberal* portion of the cabinet prevail over the *illiberal.* That this ascendancy may continue to gain strength is our sincere wish; and we rejoice at this unequivocal indication of such ascendancy.

That item of the new regulations which requires attendance on two courses of anatomical lectures, and two courses of dissections, *previously* to county hospital time being allowed, is designated as "most diabolical." These are hard words; but then we must make allowance for the intense morality of the Press. "Will any prudent parent," says this exemplary monitor, "allow his child to be on the pavé of this metropolis without a protector, at the age of fifteen or sixteen?" This reminds us of the soldier's anxiety for the safety of the church, in Goldsmith. What dreadful risks must the youth of 15 run, in this metropolis! He may be allured to his ruin by some warbling syren—or transformed into a hog by some enchanting Circé—or, what is far more likely, he may be MACADAMIZED while crossing some public thoroughfare in his way from one lecture-room to another!

None of these things can possibly happen when he has attained the mature age of 19 or 20. But as hospital practice is only a counter-part of private practice, and as many people have thought that some knowledge of anatomy, and some acquaintance with the principles of surgery, were absolutely necessary as preliminary steps for seeing, with advantage, the one, or acting with safety, in the other, we venture to say that, not only the *county* hospital time, but the metropolitan also, should be *subsequent* to a certain attendance on anatomical and surgical lectures. It would be a most edifying sight to behold a youth of 18 or 19, coming up to town with certificates of his hospital education complete, before he had performed a single dissection, or attended a single lecture! We would recommend, on the contrary, that the youth should be kept at his general education till he is 17—that the next two years be dedicated to the acquirement of pharmaceutical knowledge—that one year's study of anatomy and surgery, at least, should precede his attendance on hospitals—and that the choice of hospitals, whether in town or country, be guided by the circumstances of the individual.

---

### 62. IRON BLISTERS.

Sir Anthony Carlisle has invented a new mode of metallic blistering, which he considers to be superior, cheaper, quicker, and, on the whole, less painful than the common mode of proceeding with the emplastrum lyttæ The process consists in immersing a piece of polished iron in boiling water, for five or six minutes, and then applying it to the part which is to be blistered, interposing a piece of wetted silk. The hot instrument is to be kept on the part for three or four seconds. The pain is considerable—an inflammatory process is set up—serum is exuded—and the cuticle detached. We do not deem ourselves justified in hazarding any opinion on the comparative merits of this mode of blistering, as we have not yet put it to the test. It may be useful in hospital practice, and in private life, where great dispatch is desirable. We do not apprehend, however, that it will ever come into general use in private practice, on account of the prejudices which are entertained against instrumental operations—in short, against

——————— the perils that environ
The man that meddles with *hot* iron.

### 63. IMPORTANT ANATOMICAL DISCOVERY!

Two columns and a quarter of the LANCET are occupied with a most profound enquiry, "Whether the thumb has a metacarpal bone and two phalanges, or *no* metacarpal bone and three phalanges?" After ransacking a host of authors, from *Aristoteles* (who was a great anatomist in his day) down to Blumenbach; it is at length determined that the thumb is the longest as well as the strongest finger of the hand, although it disdains to walk upon stilts, like its four ambitious neighbours.

### 64. NEW PATENTS.

The Radical Press Company have obtained a patent for VITUPERATION, and are determined to prosecute with the utmost rigour of the *Law*, all those who dare to invade their property.

### 65. NON-JURORS—RETARDATION OF MEDICAL SCIENCE.

A luminary of the Radical Press, a *soi-disant* fellow of the College of Physicians, has discovered that the great impediment to the march of medical science is the subscription to the 39 articles of the Church of England, enjoined by the College on its members. He, therefore, calls upon the medical profession to rise, *en masse*, "physicians, surgeons, and apothecaries, either to establish a *new* and more liberal College of Physicians and examiners, or to ameliorate and rescind the offensive regulations of the old one." The Radical Press has unquestionable power to *call* spirits from the vasty deep —But, query, *will they come?*—Nous verrons. There may be defects in the College of Physicians, as in all other human institutions; but, Heaven defend us from the democracy of the Medico-Radicals! There is little danger, however, that these gentry will be listened to, either in or out of Parliament.

Non tali auxilio, nec defensoribus istis,
Tempus eget.

### 66. PREGNANCY COMPLICATED WITH HYDATIDS.

Dr. Thuillier, of Amiens, has published a case of this kind, in a late No. of the Journal General, of which we shall give a brief abstract in this place.

*Case.* Mad. Hec.—aged 40 years, had been plunged in misery and distress, from her own bad conduct, for some years. She was subject to attacks of lipothymia and vomiting after eating. For four months the menses had been wanting, and it was supposed she was pregnant. Dr. T. examined, but could not decide on this point. The woman herself, who had borne nine children, did not consider it as pregnancy. She believed, from the vomiting after food, that she laboured under scirrhus of the stomach, of which complaint her mother had died. Low diet and diluents were prescribed. There was now developed much irritation about the uterus, and leeches were applied to the groins and vagina. The irritation was removed. The stomach complaint was now much relieved. A month or more after this, our author was hastily summoned to Madame H. who was said to be in labour. The uterus was now much larger than when last examined, and seemed to fill the pelvis. There were bearing-down pains, and in these, the uterus felt very turgid. The os uteri, however, was close, and no discharge from thence. The pains persisted, without any alteration in the cervix or os uteri. This state continued three days more, when it was perceived that, at each bearing-down pain, there was some discharge of clear water. This was followed some days afterwards, by a large number of hydatids, of various sizes, from an inch in diameter downwards. She continued to pass these bodies for fifteen days. In the mean time the size of the uterus rapidly increased, and rose out of the pelvis. It was now ascertained that there was a fœtus in utero, and yet the hydatids were discharged daily in considerable quantities. Three or four months after this, she was delivered of a child at the full term, the hydatids having never ceased one day to be discharged. There were one hundred and forty-eight of these bodies collected, and the number broken and unobserved could not be estimated. The patient recovered and did well. She has since become pregnant.

---

### 67. CURE OF PHTHISIS BY MERCURY.

Our readers will be able to draw their own conclusions from the following case.

A young man, a West-Indian, 25 years of age, had suffered severe attacks of pulmonic inflammation, for which bleeding and other antiphlogistic measures were employed. After this, symptoms of phthisis were developed in a very unequivocal character, when he came under the care of Dr. Dufau, of Mont de Marsau. The symptoms need not be detailed here. The principal were copious purulent expectoration—hectic fever—perspirations—difficulty of breathing—pain in the chest—emaciation to an extreme degree. The right side of the chest emitted a very dull sound on percussion. In this condition, symptoms of constitutional syphilis appeared, and determined Dr. Dufau to put the patient on a course of sarsaparilla and the oxymuriate of mercury, as his case could hardly be rendered worse. In the course of a month, the phenomena of phthisis gradually subsided, and the young man was ultimately restored to complete health. The sound of the right side of the chest became as clear as that of the left.

The author comes to the conclusion that the phthisis, in this case, had something special in it—in short, that it was of a syphilitic character, and that, on this principle, the cure was effected by the sarsaparilla and oxymuriate.

---

### 68. NEW OPERATION FOR PILES.

Dr. J. C. Rousseau has proposed, in one of our American contemporaries, a new mode of removing those fungous excrescences around the anus, which are occasionally found to exist when piles have become permanently protruded, giving rise to troublesome ulcerations, hæmorrhage, fistulæ, &c. The mode of removal proposed by Dr. R. is by a multiplicity of ligatures, between each of which only a small portion of the excrescence is to be included. For this purpose, he arms a needle with two different coloured threads, and then thrusts it through, from the verge of the anus to the outside of the tumour, and again from this side to the former, and so on alternately until the whole bulk has been stitched all around, observing between each passage of the needle to keep a distance of about one-third of an inch, on the outside, and much less on the inside. When this process is finished, (leaving a loop of ligature loose at each pull of the needle,) the ligatures are to be divided in the loops, and then each tied to its corresponding branch. By this means the whole mass, without any intervening portion, is inclosed in ligatures.

We cannot but consider this as a tedious and somewhat difficult process, and moreover unnecessary. For where old piles are thus consolidated into external fungous excrescences, there can be little danger in removing them by knife or ligature. This, however, is not the opinion of Dr. Rousseau, and to him we must bow. At all events, this mode of operation will not do for the more difficult and dangerous removal of piles which come down only occasionally, and which are of far greater detriment to the patients' health and comfort than the external tumours.

### 69. FRACTURES OF THE CERVIX FEMORIS.

To the strong phalanx of cases brought forward by Sir Astley Cooper, proving the want of bony union in cases of fracture of the cervix femoris within the capsular membrane, Mr. Mayo has lately added another. A woman, 50 years of age, had a violent fall on her hip. The limb was not sensibly shortened, but was rendered useless. Pain and swelling succeeded, and she was confined to her bed for five months, when she regained some degree of strength in the injured hip, and gradually became enabled to walk, with the assistance of a stick. Thirteen months after this accident the woman died of apoplexy. On examination, the case was found to have been fracture of the cervix femoris within the capsular ligament. "Union had taken place by means of an intervening layer of soft but tough substance, from two to four lines in thickness; in which, however, nothing like commencing ossification was detected."

For some speculations on the causes of this non-union, we refer our readers to the 6th number, new series, of the Medical and Physical Journal.

---

### 70. INTERNAL STRANGULATION.

If dissection followed death in every case of what is called fatal enteritis, or obstruction of the intestines, several would be found to depend, not on inflammation, nor yet on accumulation in the cavity of the intestine, but on a mechanical obstruction—in fact, on an internal hernia. It is not long since we recorded a curious case of this kind, in the person of Mrs. Belzoni's servant and fellow traveller. Some cases are on record where the appendix cæci vermiformis had strangled a portion of intestine. Dr. Brown, of Sunderland, has lately mentioned a case where "the vermiform appendix had contracted an adhesion to the mesentery, and thus formed a loop, or noose, by means of which, a portion of small intestine, about two yards in length, was completely strangulated." The symptoms resembled those of strangulated hernia; but, as there was no external tumour, bleeding, purgatives, enemata, &c. were properly employed. All means failed, of course, and the foregoing phenomena presented themselves on dissection. In this case, and in that also of Mrs. Belzoni's servant, gastrotomy would, in all probability, have saved life, if performed before sphacelus had taken place. But who will be so bold as to perform this formidable operation on the *chance* of there being a mechanical strangulation capable of liberation?

---

### 71. PHLOGOSIS OF THE RETINA.

It is probable that many cases of amaurosis depend on inflammation of the retina itself, as well as on affections of the optic nerve within the head. There are instances on record where the sight was suddenly lost, by this species of phlogosis, and as suddenly restored by proper means. Two cases of this kind recently occurred in Bartholomew's Hospital, and are reported from that institution. The first was a man who went to bed without any affection of the eye, but, in the morning, found the sight of the right eye much impaired, and, in a few hours more, almost entirely lost. There was some inflammation of the conjunctiva—the pupil natural—pain in the head and eye. He was bled *ad deliquium*, and had a cathartic of calomel and jalap. After the operation of the medicine, he was directed to take calomel and opium every six hours. Next day, he was bled locally from the temples, and in another day he could read the smallest print.

The other case was nearly similar but of longer standing, and required, besides the sanguineous evacuations, some days' continuance of the calomel and opium.

In such cases the treatment should be prompt, otherwise the delicate structure of the retina may be irrecoverably disorganized.—LANCET.

